# Supplementary material for: Rapid measurement of inhibitor binding kinetics by isothermal titration calorimetry
Source: Nat Commun. 2018 Mar 1;9:893. doi: 10.1038/s41467-018-03263-3 (PMC5832847; doi:10.1038/s41467-018-03263-3)
Supplement: Supplementary file 1 — Supplementary Information [file 41467_2018_3263_MOESM1_ESM.pdf]

Supplementary Information:
Rapid Measurement of Inhibitor Binding Kinetics by Isothermal Titration Calorimetry

Justin M. Di Trani[1], Stephane De Cesco[1], Rebecca O'Leary[1], Jessica Plescia[1], Claudia Jorge do Nascimento[1,2], Nicolas Moitessier[1], Anthony K. Mittermaier*[,1]

[1]Department of Chemistry, McGill University, H3A 0B8 Montreal, Canada

[2]Institute of Biosciences, Federal University of the State of Rio de Janeiro, Urca, Rio de Janeiro, Brazil

*Fax: 514-398-3797

*Email: anthony.mittermaier@mcgill.ca

TABLE OF CONTENTS

SUPPLEMENTARY METHODS **3-14**
Prolyl oligopeptidase purification **3-4**
Baseline correction **4**
Supplementary Fig. 1. Baseline correction of raw data for inhibition experiment using compound 1 4
Blank subtraction **4-5**
Supplementary Fig. 2. Determination of $\boldsymbol{\tau}_{blank}$ for inhibition experiment using compound 1 5
Kinetics of Inhibition Model **5-6**
Covalent Inhibition **6-7**
ITC kinetics fitting Scripts **7-11**
Global fits for ITC kinetics **11**
Spectroscopy Fitting Scripts **11-12**
Prolyl oligopeptidase enthalpy of catalysis and Michaelis-Menten parameters **13**
Supplementary Fig. 3. Prolyl oligopeptidase and TRH enthalpy of catalysis 13
Supplementary Fig. 4. Michaelis-Menten plot for prolyl oligopeptidase with TRH 13
Supplementary Table 1. Summary of prolyl oligopeptidase enzyme parameters with TRH 14
Enthalpy of binding in kinetics of inhibition and initiation experiments **14-15**
Supplementary Fig. 5. Compound 5 prolyl oligopeptidase binding experiments 14
Supplementary Table 2. Summary of compound 5 prolyl oligopeptidase binding parameters 15
Supplementary Fig. 6. Modelling heat of binding in kinetics of inhibition experiments 15

SUPPLEMENTARY NOTE 1 - RESULTS **16-28**
Fits for ITC kinetics of inhibition and initiation experiments **16-21**
Supplementary Fig. 7. Kinetics of inhibition experiment using compound 1 16
Supplementary Fig. 8. Kinetics of initiation experiment using compound 1 16
Supplementary Fig. 9. Global fit for compound 1 17
Supplementary Fig. 10. Kinetics of inhibition experiment using compound 2 17
Supplementary Fig. 11. Kinetics of initiation experiment using compound 2 18
Supplementary Fig. 12. Global fit for compound 2 18
Supplementary Fig. 13. Kinetics of inhibition experiment using compound 3 19
Supplementary Fig. 14. Kinetics of inhibition experiment using compound 4 19
Supplementary Fig. 15. Kinetics of initiation experiment using compound 4 20
Supplementary Fig. 16. Global fit for compound 4 20
Supplementary Fig. 17. Kinetics of inhibition experiment using compound 5 21
UV-Vis and NMR Spectroscopy experiments **21-22**
Supplementary Fig. 18. UV-Vis inhbtion experiment using compound 1 21
Supplementary Fig. 19. UV-Vis initiation experiments using compound 1 22
Supplementary Fig. 20. NMR inhibtion experiment using compound 5 22
Statistical analysis of errors **23-28**
Supplementary Fig. 21. Confidence level contour plots for ITC experiments using compound 1 24
Supplementary Fig. 22. Confidence level contour plots for UV-VIS experiments using compound 1 25

Supplementary Fig. 23. Confidence level contour plots for ITC experiments using compound 2 25
Supplementary Fig. 24. Confidence level contour plots for ITC experiments using compound 3 26
Supplementary Fig. 25. Confidence level contour plots for ITC experiments using compound 4 27
Supplementary Fig. 26. Confidence level contour plots for ITC experiments using compound 5 28
Supplementary Fig. 27. Confidence level contour plots for ITC experiments using compound 5 28
Supplementary Table 3. Kinetic and thermodynamic parameters 28

SUPPLEMENTARY NOTE 2 – GENERAL CONSIDERATIONS 29-43
1 - Introduction 29
2 - Instrument parameters 29-30
2.1 - Pre injection delay 29
2.2 - Injection volume 29
2.3 - Spacing between injections 30
3 - Kinetic limitations 30-33
3.1 - Upper kinetic rate limits 30-32
Supplementary Fig. 28. Monte Carlo simulations of first order kinetics experiments 31
Supplementary Fig. 29. Experimental confirmation of SNR_{min} 32
3.2 - Lower kinetic rate limits 32-33
Supplementary Fig. 30. Q_{RMS} vs. τ 33
4 - Kinetics of inhibition experiments 34-37
4.1 – Measuring thermodynamics and kinetic properties for an enzyme-substrate pair 34
4.2 - Enzyme and substrate concentrations 34-35
4.2.1 - Maximizing signal in inhibition experiments 34
4.2.2 - Steady rate enzyme kinetics in inhibition experiments 34-35
4.2.3 - c-value and its implications for experimental design 35
4.3 - Inhibitor concentration 35-36
4.3.1 - Tailoring inhibition kinetics 35-36
4.3.2 - Decreasing inhibitor concentration 36
4.3.3 - Increasing inhibitor concentration 36
4.4 – Limitations for the K_i 36-37
4.4.1 - Upper limit for K_i. 36-37
4.4.2 - Monte Carlo simulations for the lower K_i limit 37-38
Supplementary Fig. 31. Monte Carlo simulations of inhibition experiments 38
4.5 – Setting up inhibition experiments 38-39
5 - Kinetics of initiation experiments 39-43
5.1 - Measuring thermodynamics and kinetic properties for an enzyme-substrate pair 39
5.2 - Enzyme and substrate concentrations 39-40
5.2.1 - Maximizing signal in initiation experiments 39
5.2.2 - Steady rate enzyme kinetics in initiation experiments 39
5.2.3 - Substrate concentration 39-40
5.3 - Limitations for the K_i 40-41

5.3.1 - Upper limit for K_i 40
5.3.2 - Monte Carlo simulations for the lower K_i limit 40-41
Supplementary Fig. 32. Monte Carlo simulations of initiation experiments 41
5.4 - Optimizing signal due to dissociation 41-42
5.4.1 – Dissociation of enzyme-inhibitor complex due to dilution 41-42
5.5 - Setting up initiation experiments 42
6 – Data analysis workflow 42-43

SUPPLEMENTARY REFERENCES 43-44

SUPPLEMENTARY METHODS

Prolyl oligopeptidase purification. Protein Expression. E. coli BL21 competent cells were transformed with pETM10 hPOP. A starter culture of LB medium (100 mL) containing kanamycin (50 mg mL^{-1}) was inoculated with one colony and was incubated overnight at 37 °C with shaking. After 16 h, four cultures of LB (4 × 1000 mL) containing kanamycin (50 mg mL^{-1}) were inoculated with the overnight culture (20 mL). The inoculated cultures were incubated at 37 °C and 220 rpm until the OD600 was between 0.3 and 0.5 (3 h). The temperature was lowered to 18 °C, after one hour of temperature equilibration IPTG was added (final concentration of 0.5 mM), and induction was allowed to proceed for 5 h. Cells were harvested by centrifugation (4000g, 15 min, 4 °C), and the pellet was resuspended in suspension buffer (50 mL) [Tris-HCl (10 mM), NaCl (300 mM), β-mercaptoethanol (5 mM), imidazole (1 mM), and 5% glycerol, pH 8] and sonicated for four cycles (2 min of sonication/2 min of rest; pulse, 0.5 intensity; duty, 0.5), while the sample was kept on ice (Branson sonifier 450, Emerson industrial automation, United States). After sonication, the sample was centrifuged (40000g, 30 min, 4 °C), and the supernatant was used immediately for POP purification. An affinity column was used (10 mL, Toyopearl, AF-Chelate-650M) for purification. The supernatant was applied at a flow rate of 0.5 mL min−1 to a column previously equilibrated with 2 column volumes of NiSO4 (0.2 M) followed by 5 column volumes of suspension buffer. The column was then washed with 5 column volumes of suspension buffer, then 5 column volumes of washing buffer [Tris-HCl (20 mM), NaCl (300 mM), β-mercaptoethanol (5 mM), imidazole (15 mM), and 5% glycerol, pH 8]. The elution was then performed with 4 column volumes of elution buffer [Tris-HCl (20 mM), NaCl (300 mM), β-mercaptoethanol (5 mM), imidazole (500 mM), and 5% glycerol, pH 8]. Fractions (4 mL) were collected during the entire elution. Fractions testing positive for POP activity were analyzed by SDS−PAGE and stained with phastgel blue R (GE Healthcare, sweden). POP-containing fractions were combined and subjected to size exclusion chromatography (HiLoad 16/60 Superdex75 prep grade on a GE Healthcare Äkta Avant system) with [Tris-HCl (20 mM), NaCl (150 mM), benzamidine (5 mM), EDTA (1 mM), β-

mercaptoethanol [5 mM], and 5% glycerol, pH 8] as the running buffer. The purified enzyme was dialyzed into the appropriate buffer. Recombinant hPOP was quantified by measuring the absorbance at 280 nm using an extinction coefficient calculated by the following equation (ε = nTrp*5000 + nTyr*1490 + nCys*125, 129090 $L\cdot mol^{-1}\cdot cm^{-1}$ for POP).

Baseline correction. In order to correct for sloped baselines, a common artifact in ITC experiments, a baseline correction procedure was implemented for both kinetics of inhibition and initiation experiments. This procedure involves fitting lines to the final flat (usually last ~200 s) portion of each injection (**Supplementary Fig. 1a**). Each of these lines is extrapolated back to the beginning of the respective injection in order to establish a full baseline for each injection. The full baseline is then subtracted from the raw data for each injection to give the baseline-corrected data (**Supplementary Fig. 1b**).

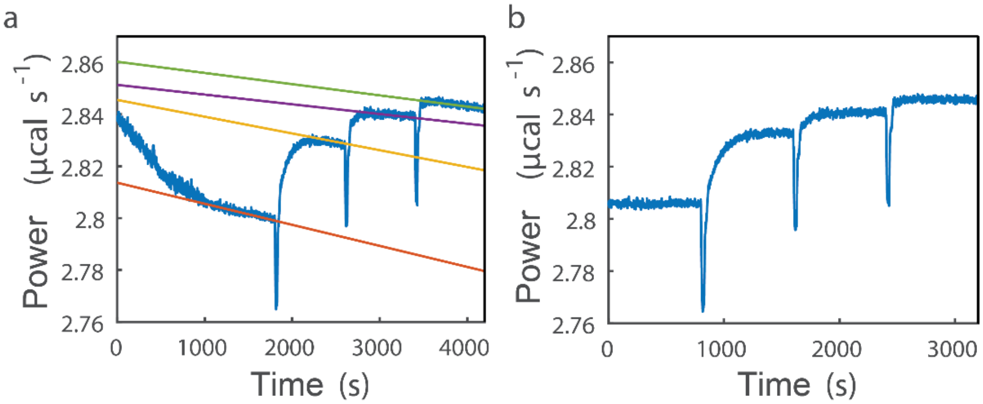

Supplementary Figure 1. Baseline correction of raw data for inhibition experiment using compound 1. a) Raw data including pre injection delay (0-1000 s) and 4 injections (1st injection 0.4 µL over 0.8 s, subsequent 3 injections 13 µL over 26 s) of compound **1** (7 µM in syringe) into cell containing POP and TRH (1.32 nM and 13 mM) using a Malvern ITC-200 (blue line). Orange, yellow, purple and green lines show the baselines for injections 1, 2 3 and 4 respectively. b) Baseline corrected ITC kinetics of inhibition data.

Blank subtraction. ITC injections are accompanied by heat produced due to the dilution of the contents of the syringe into the cell as well as the mechanical injection process itself. This heat is detected during and immediately following the injection, partially obscuring the desired signal for an amount of time that depends on the response function of the calorimeter. We have found that the most robust way to

circumvent these injection artifacts is to employ a blank experiment performed identically to the actual experiment except without either no enzyme or no substrate. The blank experiment is used to determine when the injection artifact ends (**τ_{blank}**) and is used as the starting point for the data analysis of each injection (see ITC kinetics fitting scripts). Note that τ_{blank} is approximately given by τ_{inj} + 3×τ_r, where τ_{inj} is the length of the injection and τ_r is the empirical calorimeter response time (≈10 s for a Malvern ITC-200 and ≈20 s for a Malvern VP-ITC), although the best estimate is obtained directly from the blank injections (**Supplementary Fig. 2**).

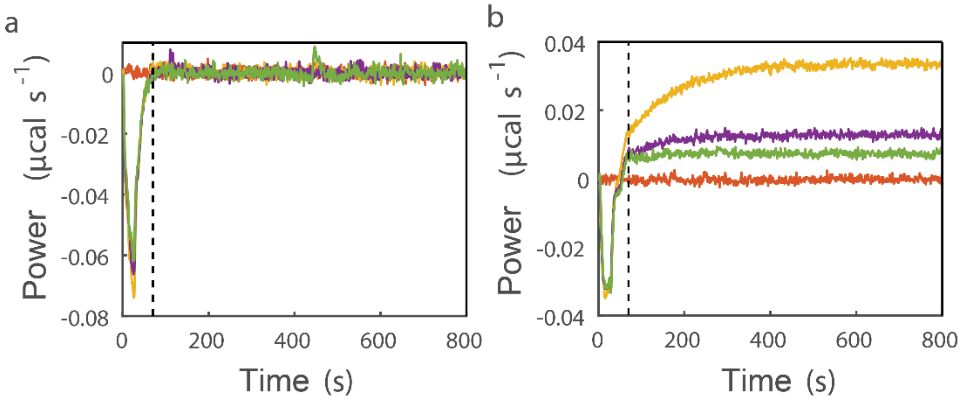

Supplementary Figure 2. Determination of τ_{blank} for inhibition experiment using compound 1. a) Blank experiment injections for inhibition experiment of compound **1**. This experiment is performed under exactly the same conditions as actual experiment except with no POP. **τ_{blank}** is determined to be time it takes the signal to return to baseline after an injection (dashed line at 70 s). b) Overlay of injections from inhibition experiment of compound **1** black dashed line represents the value for **τ_{blank}**.

Kinetics of inhibition model. Simple competitive enzyme inhibition is described by the following kinetic scheme

$$\mathrm{EI} \underset{k_{on}}{\overset{k_{off}}{\rightleftharpoons}} \mathrm{E} + \mathrm{I} + \mathrm{S} \underset{k_{-1}}{\overset{k_1}{\rightleftharpoons}} \mathrm{ES} \overset{k_{cat}}{\rightarrow} \mathrm{E} + \mathrm{P} \qquad (1)$$

where E, S, P, and I represent the enzyme, substrate, product, and inhibitor, respectively. The potency of an inhibitor is typically described by its inhibition constant, $K_i = k_{off}/k_{on}$, or by the concentration required to achieve 50% steady-state inhibition at a given substrate concentration, $IC_{50} = K_i(1+[S]/K_m)$, where K_m is

the Michaelis constant, $K_m = (k_{-1}+k_{cat})/k_1$.[1] Although measuring these equilibrium parameters represents a key step in drug optimization, it is also increasingly of interest to know how quickly the enzyme loses activity once an inhibitor is added and how quickly activity is restored if the free inhibitor is removed, as discussed above. These dynamic processes are largely governed by the association rate, k_{on}, and the disassociation rate k_{off}, which is the inverse of the residence time, $\tau_R=1/k_{off}$. The instantaneous rate of enzyme catalysis is given by the Michaelis-Menten equation

$$\frac{d}{dt}[P] = k_{cat}[ES] = \frac{k_{cat}([E]_{tot} - [EI])[S]}{K_m + [S]} \tag{2}$$

while the kinetics of inhibition are given by

$$\frac{d}{dt}[EI] = k_{on}[E][I] - k_{off}[EI] \tag{3}$$

Typical enzyme assays employ spectroscopic,[1, 2] chromatographic,[3, 4] or electrophoretic,[4] techniques to monitor the concentrations of products or substrates, thereby yielding rates of catalysis. To measure K_i or IC_{50} values, the enzyme is allowed equilibrate thoroughly with an inhibitor, such that [EI] can be considered time-invariant, depending only on the amount of inhibitor added. To characterize k_{on} or k_{off}, the pre-equilibration time with the inhibitor is varied,[5] or substrate and product concentrations are measured while [EI] gradually changes due to inhibitor association or dissociation.[6]

Covalent Inhibition. One complication of covalent inhibitors is that their binding mechanisms are usually considered to have at least two distinct steps.[1, 2, 7, 8] The enzyme and inhibitor first interact non-covalently (EI) and subsequently form a covalent bond (E-I), according to **equation 4**.

$$\mathrm{E + I} \underset{k_{-n}}{\overset{k_n}{\rightleftharpoons}} \mathrm{EI} \underset{k_{-c}}{\overset{k_c}{\rightleftharpoons}} \mathrm{E - I} \tag{4}$$

where k_n and k_{-n} are second and first order rate constants describing the non-covalent association and dissociation of the inhibitor, while k_c and k_{-c} are first order rate constants describing formation and breakage of the covalent bond, respectively. When (k_{-n} + k_c) >> $k_n[I]$, EI does not accumulate appreciably as a kinetic intermediate and the kinetics of inhibition are indistinguishable from those of simple non-covalent inhibition.[2, 4] Formation of the inhibited state (EI + E-I) follows **equation 3** with an apparent association rate constant:

$$k'_{on} = \frac{k_n k_c}{k_c + k_{-n}} \tag{5}$$

When this kinetic condition is not met, bi-phasic inhibition is obtained, characterized by rapid formation of EI followed by gradual conversion to E-I.[2, 7] Similarly, when k_c>>k_{-c} or k_c>>k_{-n} dissociation is kinetically first order with an apparent rate constant:

$$k'_{off} = \frac{k_{-n} k_{-c}}{k_c + k_{-n}} \tag{6}$$

When k_{-n}>>k_c≈k_{-c}, dissociation is biphasic with rapid dissociation of EI followed by gradual loss of E-I. In some cases, biphasic inhibition kinetics are observed experimentally, giving information on the microscopic parameters in **Scheme 2**.[9, 10] In many cases however, binding is empirically monophasic and the apparent association and dissociation represent the experimentally-accessible physical parameters.

ITC kinetics fitting Scripts. All fitting routines were performed using in-house MATLAB scripts. Differential equations were integrated numerically in 0.01s integration steps. For kinetics of inhibition experiments:

$$[ES]_{t,i} = \frac{[S]_{t,i} * ([E_o]_{t,i} - [EI]_{t,i})}{[S]_{t,i} + K_m} \tag{7}$$

$$\frac{d[P]_{t,i}}{dt} = k_{cat} * [ES]_{t,i} \tag{8}$$

$$[E]_{t,i} = [E_o]_{t,i} - [EI]_{t,i} - [ES]_{t,i} \tag{9}$$

$$\frac{d[EI]_{t,i}}{dt} = k_2 * [E]_{t,i} * [I]_{t,i} - k_{-2} * [EI]_{t,i} - [EI]_{t,i} * R_{dil,i} \quad (10)$$

$$[EI]_{t+dt,i} = \frac{d[EI]_{t,i}}{dt} * dt + [EI]_{t,i} \quad (11)$$

$$\frac{d[I]_{t,i}}{dt} = -k_2 * [E]_{t,i} * [I]_{t,i} + k_{-2} * [EI]_{t,i} + [I]_{syr} * R_{dil,I} - [I]_{t,i} * R_{dil,i} \quad (12)$$

$$[I]_{t+dt,i} = \frac{d[I]_{t,i}}{dt} * dt + [I]_{t,i} \quad (13)$$

$$\frac{d[S]_{t,i}}{dt} = -[S]_{t,i} * R_{dil,i} - \frac{d[P]_{t,i}}{dt} \quad (14)$$

$$[S]_{t+dt,i} = \frac{d[S]_{t,i}}{dt} * dt + [S]_{t,i} \quad (15)$$

$$\frac{d[E_o]_{t,i}}{dt} = -[E_o]_{t,i} * R_{dil,i} \quad (16)$$

$$[E_o]_{t+dt,i} = \frac{d[E_o]_{t,i}}{dt} * dt + [E_o]_{t,i} \quad (17)$$

For kinetics of initiation experiments:

$$[ES]_{t,i} = \frac{[S]_{t,i} * ([E_o]_{t,i} - [EI]_{t,i})}{[S]_{t,i} + K_m} \quad (18)$$

$$\frac{d[P]_{t,i}}{dt} = k_{cat} * [ES]_{t,i} \quad (19)$$

$$[E]_{t,i} = [E_o]_{t,i} - [EI]_{t,i} - [ES]_{t,i} \quad (20)$$

$$\frac{d[EI]_{t,i}}{dt} = k_2 * [E]_{t,i} * [I]_{t,i} - k_{-2} * [EI]_{t,i} - [EI]_{t,i} * R_{dil,i} + [EI]_{syr} * R_{dil,i} \quad (21)$$

$$[EI]_{t+dt,i} = \frac{d[EI]_{t,i}}{dt} * dt + [EI]_{t,i} \quad (22)$$

$$\frac{d[I]_{t,i}}{dt} = -k_2 * [E]_{t,i} * [I]_{t,i} + k_{-2} * [EI]_{t,i} + [I]_{sry} * R_{dil,i} - [I]_{t,i} * R_{dil,i} \quad (23)$$

$$[I]_{t+dt,i} = \frac{d[I]_{t,i}}{dt} * dt + [I]_{t,i} \quad (24)$$

$$\frac{d[S]_{t,i}}{dt} = -[S]_{t,i} * R_{dil,i} - \frac{d[P]_{t,i}}{dt} \quad (25)$$

$$[S]_{t+dt,i} = \frac{d[S]_{t,i}}{dt} * dt + [S]_{t,i} \quad (26)$$

$$\frac{d[E_o]_{t,i}}{dt} = -[E_o]_{t,i} * R_{dil,i} \quad (27)$$

$$[E_o]_{t+dt,i} = \frac{d[E_o]_{t,i}}{dt} * dt + [E_o]_{t,i} \quad (28)$$

For both inhibition and initiation experiments the k_{cat} and K_m are the catalytic rate and the Michaelis-Menten constants for TRH respectively. $[E_o]_{t,i}$ is the total concentration of enzyme for time = t for injection i and $[I]_{t,i}$, $[E]_{t,i}$, $[S]_{t,i}$, $[EI]_{t,i}$, and $[ES]_{t,i}$ are the concentrations of free inhibitor, free enzyme, free substrate, enzyme inhibitor complex, and enzyme substrate complex in the cell at time = t for the ith injection respectively. k_2 and k_{-2} are the association rate dissociation rate of the inhibitor respectively. The dilution rate ($R_{dil,i}$) is the rate of dilution occurs for injection i; this is equal to dV/dt*1/V_{cell} during the injection and 0 otherwise. Here dV/dt is the rate of injection and V_{cell} is the volume of the cell.

For ITC inhibition experiments $[I]_{syr}$ is the concentration of inhibitor in the syringe. The initial conditions for the first injection (i = 1) are $[E_o]_{t=1,i=1} = [E_o]_{cell}$, $[I]_{t=1,i=1}$ =0, $[S]_{t=1,I,=1} = [S]_{cell}$ and $[EI]_{t=1,i=1}$ =0. For subsequent injections (i > 1) the initial conditions were set as $[E_o]_{t=1,i} = [E_o]_{t=\,ttot,i-1}$, $[I]_{t=1,i} = [I]_{t=\,ttot,i-1}$, $[S]_{t=1,i} = [S]_{t=\,ttot,i-1}$ and $[EI]_{t=1,i} = [EI]_{t=ttot,i-1}$ where t_{tot} is the total time between injections and $[E_o]_{cell}$ / $[S]_{cell}$ are the initial concentrations of enzyme/substrate in the cell (ie. i=1, t=0).

For ITC initiation experiments $[EI]_{syr}$, $[I]_{syr}$ and $[E]_{syr}$ are calculated via the binding polynomial using the total concentrations of enzyme and inhibitor in the syringe and the strength of the inhibitor $K_i = k_{-2}/k_2$.[11] The initial conditions for the first injection (i = 1) are $[E_o]_{t=1,i=1}$ = 0, $[I]_{t=1,i=1}$ =0, $[S]_{t=1,i=1} = [S]_{cell}$ and $[EI]_{t=1,i=1}$ =0. For subsequent injections (i > 1) the initial conditions were set as $[E_o]_{t=1,i} = [E_o]_{t=ttot,i-1}$, $[I]_{t=1,i} = [I]_{t=ttot,i-1}$, $[S]_{t=1,i}$ = $[S]_{t=ttot,i-1}$ and $[EI]_{t=1,i} = [EI]_{t=ttot,i-1}$ where t_{tot} is the total time between injections and $[E_o]_{cell}$ / $[S]_{cell}$ are the initial concentrations of enzyme/substrate in the cell.

The instantaneous heat of injection $h_i(t)$ is calculated using the enthalpy of the reaction ΔH_{cat} and the total volume of the cell according to:

$$h_i(t) = \Delta H_{cat} * V_{cell} * \frac{d[P]_{t,i}}{dt} \tag{29}$$

Prior to convolution the offset is adjusted such that the $h_i(t = 0) = 0$ and the initial portion of each transient is zero padded with ~500s in order to avoid artifacts from the convolution. The instantaneous heat curve is then numerically convoluted with an empirical response function[12] according to:

$$g_i(t) = h_i(t) \otimes f(t) \tag{30}$$

Where f(t) is the empirical response function, $g_i(t)$ is the resulting calculated signal, and the convolution is defined according to:

$$h_i(t) \otimes f(t) \;\; = \int_o^t f(\tau) h_i(t-\tau)\, d\tau \tag{31}$$

The calculated instrument output, $g_i(t)$, was digitally resampled in second intervals to match the experimental calorimeter output. For all compound the k_2 and K_i were floated along with ΔH_{cat} in order to account for inaccuracies in the enzyme concentration. In the case of compound **3** a stoichiometric N value was also floated (ie. $[E_o]_{cell;actual} = N*[E_o]_{cell}$ as in ordinary binding ITC experiments) while minimizing the target function (**equation 32**) using the MATLAB fminsearch function according to:

$$RSS \; = \sum_{i=1}^{M} \sum_{t=\tau_{blank}}^{t_{tot}} (g_i(t) - b_i(t))^2 \tag{32}$$

Where $b_i(t)$ is the experimental curve generated by injection i. Note that that in most experiments the first injection was omitted from the fitting (ie. *i* would start at 2 in equation 32 above) due to diffusion from the syringe tip. t_{tot} is the time between injection, M is the total number injections and $\boldsymbol{\tau}_{balnk}$ is the

beginning of the data analysis period which is determined using blank experiments (see **baseline correction and blank subtraction**).

Global fits for ITC kinetics. Global fits fitting routines were performed as above except with a global set of kinetic parameters. The target functions were calculated according to:

$$RSS_{total} = \frac{RSS_{inhib}}{(\frac{1}{t_{tot} - \tau_{blnk}} \sum_{t=\tau_{blnk}}^{t_{tot}} b_{1,inhib}(t))^2} + \frac{RSS_{init}}{(\frac{1}{t_{tot} - \tau_{blnk}} \sum_{t=\tau_{blnk}}^{t_{tot}} b_{1,init}(t))^2} \quad (33)$$

Spectroscopy Fitting Scripts. All fitting routines were performed using in house MATLAB scripts. Differential equations were integrated numerically in 0.01s integration steps. For spectroscopic kinetics of inhibition and initiation experiments:

$$[ES]_t = \frac{[S]_t * ([E_o]_t - [EI]_t)}{[S]_t + K_m} \quad (34)$$

$$\frac{d[P]_t}{dt} = k_{cat} * [ES]_t \quad (35)$$

$$[E]_t = [E_o]_t - [EI]_t - [ES]_t \quad (36)$$

$$\frac{d[EI]_t}{dt} = k_2 * [E]_t * [I]_t - k_{-2} * [EI]_t \quad (37)$$

$$[EI]_{t+dt} = \frac{d[EI]_t}{dt} * dt + [EI]_t \quad (38)$$

$$\frac{d[I]_t}{dt} = -k_2 * [E]_t * [I]_t + k_{-2} * [EI]_t \quad (39)$$

$$[I]_{t+dt} = \frac{d[I]_t}{dt} * dt + [I]_t \quad (40)$$

$$\frac{d[S]_t}{dt} = - \frac{d[P]_t}{dt} * dt \quad (41)$$

$$[S]_{t+dt} = \frac{d[S]_t}{dt} * dt + [S]_t \quad (42)$$

$$[P]_{t+dt} = \frac{d[P]_t}{dt} * dt + [P]_t \quad (43)$$

Where $[E_o]_{t,i}$ is the total concentration of enzyme at time = t for and $[I]_t$, $[E]_t$, $[S]_t$, $[P]_t$, $[EI]_t$, $[ES]_t$ are the concentrations of free inhibitor, free enzyme, free substrate, product, enzyme inhibitor complex, and enzyme substrate complex in the well plate at time = t. k_2 and k_{-2} are the association rate dissociation rate of the inhibitor respectively. The initial concentrations of the various species were used as the initial conditions for the fitting scripts. For both UV-Vis inhibition and initiation experiments the k_{cat} and K_m are the catalytic rate and the Michaelis-Menten constant respectively for ZPG-pN. For NMR spectroscopy experiments the Michaelis-Menten constants for TRH were used.

For both NMR and UV-VIs spectroscopy experiments the product concentration $[P]_t$ was digitally resampled in order to match the experimental output. For UV-Vis spectroscopy the k_2, $K_{i,}$ enzyme concentration, and intercept y_0 were floated while minimizing the target function (**equation 44**) using the fminsearch function. according to:

$$RSS = \sum_{t=0}^{t_{tot}} ([P]_{t,exp} - ([P]_{t,calc} + y_0))^2 \quad (44)$$

Where $[P]_{t,exp}$, and $[P]_{t,exp}$ are the experimental and calculated concentration of product at time t = 0 respectively. For NMR spectroscopy experiments the substrate concentration $[S]_t$ was digitally resampled in order to match the experimental output. The values of $k_{2,}$ y_0 as well as a conversion factor S_c were floated while minimizing the target function (**equation 45**) using the MATLAB fminsearch function according to:

$$RSS = \sum_{t=0}^{t_{tot}} (Sc * [S]_{t,exp} - ([S]_{t,calc} + y_0))^2 \quad (45)$$

Prolyl oligopeptidase enthalpy of catalysis and Michaelis-Menten parameters.

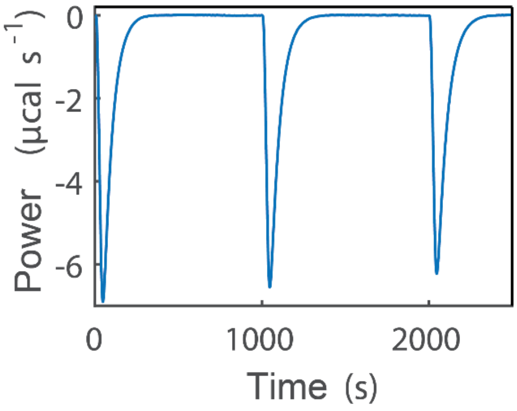

Supplementary Figure 3. **Prolyl oligopeptidase and TRH enthalpy of catalysis.** 3 injections (15 µL over 30

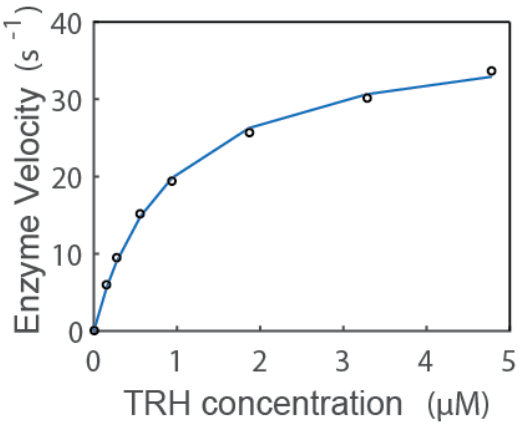

s) of Thyrotropin-releasing Hormone (TRH) (5.5 mM in syringe) into cell containing POP (250 nM in Cell) at 30°C in buffer described above. Experiment was carried out using a Malvern VP-ITC.

Supplementary Figure 4. Michaelis-Menten plot for prolyl oligopeptidase with TRH. 5 injections (8 µL, 6 µL, 15 µL, 20 µL, 50 µL, 80 µL and 90 µL over 16 s, 12 s, 30 s, 40 s, 100 s, 160 s, 180 s respectively) of Thyrotropin-releasing Hormone (TRH) (27.6 mM in syringe) into cell containing POP (2.5 nM in Cell) at 30°C in buffer described above. The enzyme velocity at several concentrations of substrate (black open circles) was extracted using the methods described in[13] and fit to Michaelis-Menten kinetics (blue curve). Experiment was carried out in Malvern VP-ITC.

Supplementary Table 1. Summary of prolyl oligopeptidase enzyme parameters with TRH. Determined by ITC using techniques presented by Gomez et al.[13]

**ΔH_{cat}**	-6.72 ± 0.06 Kcal mol^{-1}
**k_{cat}**	42.19 s^{-1}
**K_m**	975 µM

Enthalpy of binding in kinetics of inhibition and initiation experiments. In the kinetics of inhibition and initiation experiments performed here, the instrument measures both the heat generated by enzymatic catalysis and the heat produced due direct interaction of the inhibitor with the enzyme. In order to estimate the relative magnitudes of these two effects, the enthalpy of binding was directly measured using an ordinary ITC binding experiment at a concentration of POP significantly higher (approx. 100 to 1000-fold greater, 5 µM) than those used in kinetics of inhibition and initiation experiments (**Supplementary Fig. 5**). Binding data were then modelled at the concentrations of enzyme and inhibitor and injection size and spacing used for the kinetic experiment for compound **5,** with representative instrumental noise estimated from the baselines, ΔH_{bind} taken from Supplementary Table 2 and K_i taken as a previously reported value (0.5 nM)[14] (**Supplementary Fig. 6**).

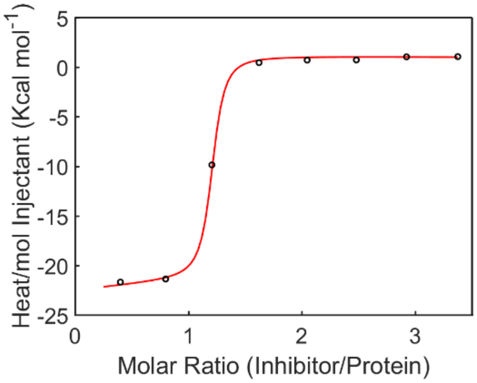

Supplementary Figure 5. Compound 5 prolyl oligopeptidase binding experiments. Compound **5** (90 µM) was titrated into 5 µM POP in cell using a Malvern ITC-200 calorimeter. First injection is 0.1µL (not shown) and subsequent 8 injections are 4.3 µL. Molar ratio of compound **5**/POP vs Total Heat for each injection peak (open black circles) with fit (red line).

Supplementary Table 2. Summary of compound **5** prolyl oligopeptidase binding parameters.

**ΔH_{bind}**	-22.2 ± 0.1 Kcal mol^{-1}
**N**	0.93 ± 0.03
**K_i**	--

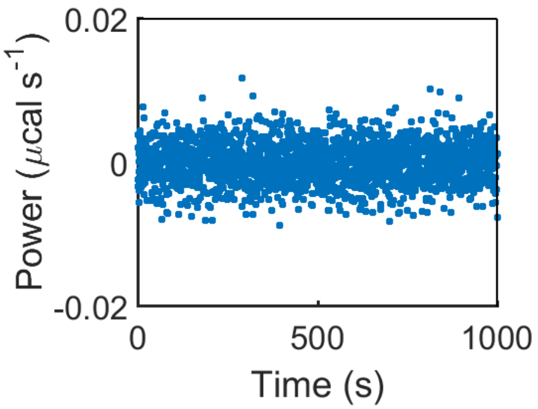

Supplementary Figure 6. Modelling heat of binding in kinetics of inhibition experiments. Modelled kinetics of inhibition experiment with compound **5** using only contributions from the heats of binding.

It is apparent that at the concentrations used in this study the heats of binding are negligible and can be omitted from the analysis. We estimate that heats of binding only become significant (>2x the noise) if either the binding enthalpy or POP concentration are over 200-fold greater than what was measured/used in this study.

SUPPLEMENTARY NOTE 1 - RESULTS

Fits for ITC kinetics of inhibition and initiation experiments

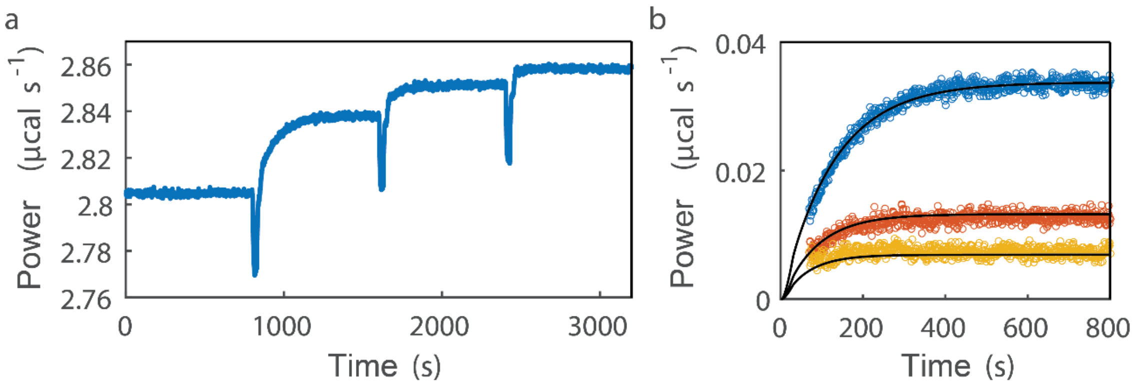

Supplementary Figure 7. Kinetics of inhibition experiment using compound 1. a) Baseline corrected data. 4 injections (1st injection 0.4 μL over 0.8 s, subsequent 3 injections 13 μL over 26 s) of compound **1** (7 μM in syringe) into cell containing POP and TRH (1.32 nM and 13 mM) at 30ºC in buffer described above using a Malvern ITC-200. a) Overlay of injections 2 (open blue circles), 3 (open orange circles), and 4 (open yellow circles) from baseline corrected ITC kinetics of inhibition data. Fit using the kinetics of inhibition model described above (black lines). Only fitted portion of the experimental curves are shown.

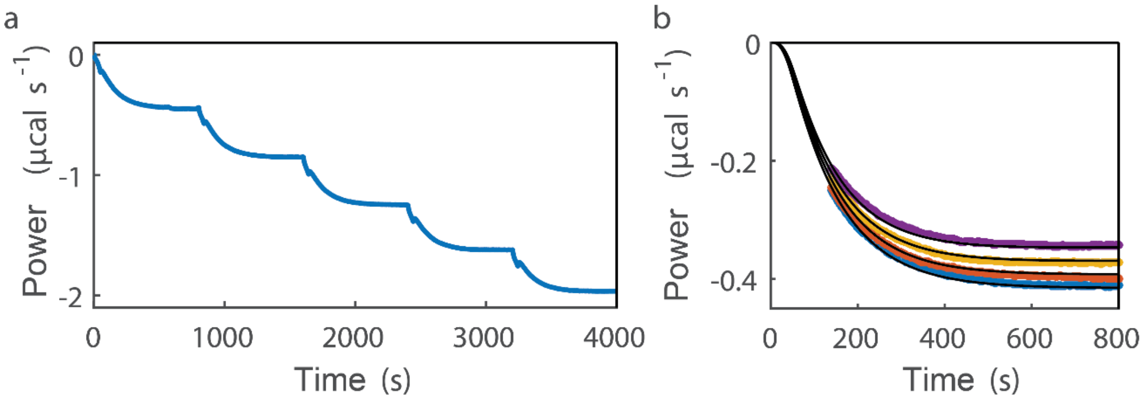

Supplementary Figure 8. Kinetics of initiation experiment using compound 1. a) Baseline corrected data, 6 injections (1st injection 25 μL over 50 s, subsequent 3 injections 20 μL over 40 s) of pre-incubated POP and compound **1** (100 nM and 878 nM respectively in syringe) into the cell containing TRH (11 mM) at 30ºC in buffer described above using a Malvern VP-ITC. b) Overlay of injections 2 (open blue circles), 3 (open orange circles), 4 (open yellow circles), 5 (open purple circles) from baseline corrected ITC kinetics of inhibition data. Fit using kinetics of inhibition model described above (black lines). Only fitted portion of the experimental curves are shown.

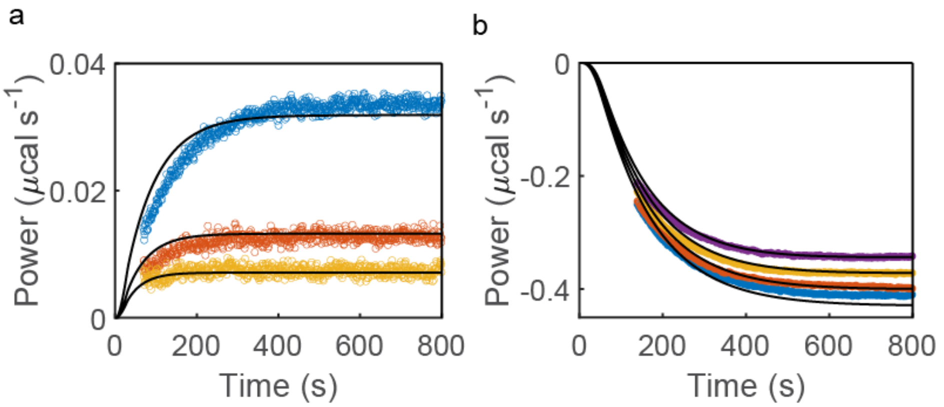

Supplementary Figure 9. Global fit for compound 1. Results of global fit between (a) inhibition experiment and (b) initiation experiment for compound **1**.

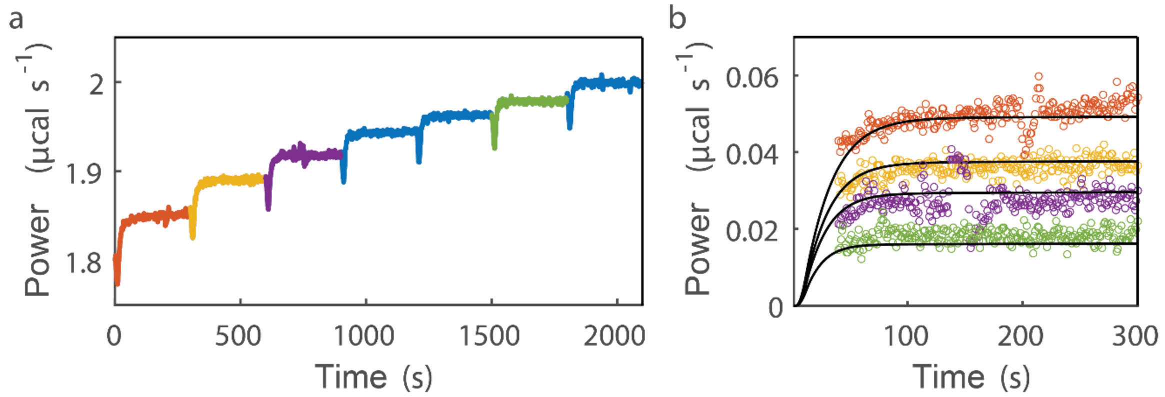

Supplementary Figure 10. Kinetics of inhibition experiment using compound 2. a) Baseline corrected data. 7 injections (all 4.5 µL over 9 s) of compound **2** (87.5 µM in syringe) into cell containing POP and TRH (5.4 nM and 18 mM) at 30°C in buffer described above using a Malvern ITC-200. b) Overlay of injections 1 (open orange circles), 2 (open yellow circles), 3 (open purple circles) and 6 (open green circles) from baseline corrected ITC kinetics of inhibition data. Fit using the kinetics of inhibition model described above (black lines). Only 4 curves are shown for clarity only, all injections except the first was used in the fitting routine. Only fitted portion of the experimental curves are shown.

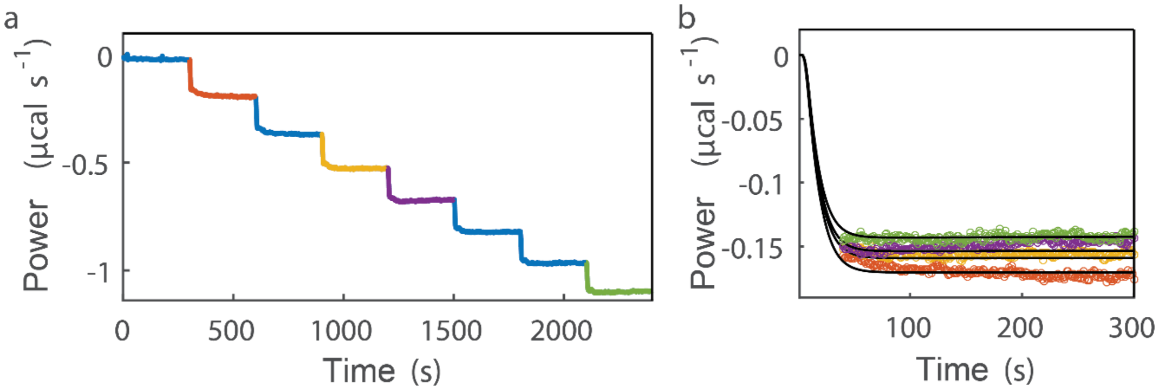

Supplementary Figure 11. Kinetics of initiation experiment using compound 2. a) Baseline corrected data, 8 injections (1st injection 0.2 µL over 0.4 s, subsequent 7 injections 2 µL over 4 s) of pre-incubated POP and compound **2** (200 nM and 16.2 µM respectively in syringe) into the cell containing TRH (17.3 mM) at 30°C in buffer described above using a Malvern ITC-200. b) Overlay of injections 2 (open blue circles), 3 (open orange circles), 4 (open yellow circles), 5 (open purple circles) from baseline corrected ITC kinetics of inhibition data. Fit using kinetics of inhibition model described above (black lines). Only fitted portion of the experimental curves are shown.

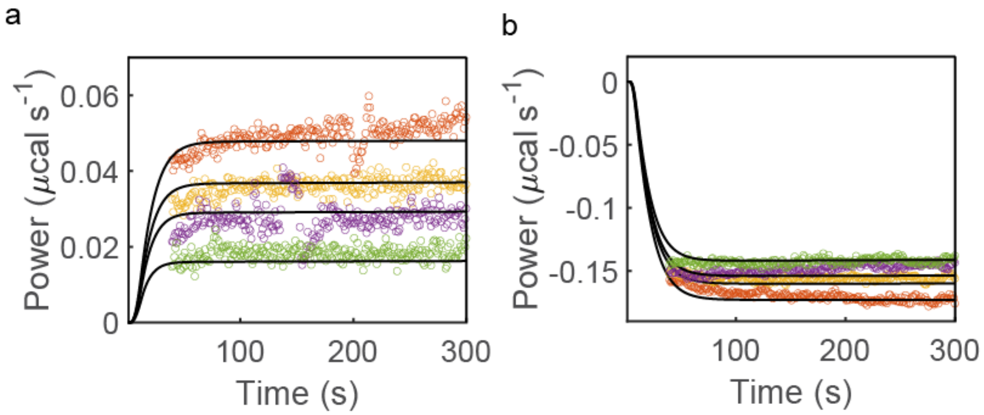

Supplementary Figure 12. Global fit for compound 2. Results of global fit between (a) inhibition experiment and (b) initiation experiment for compound **2**.

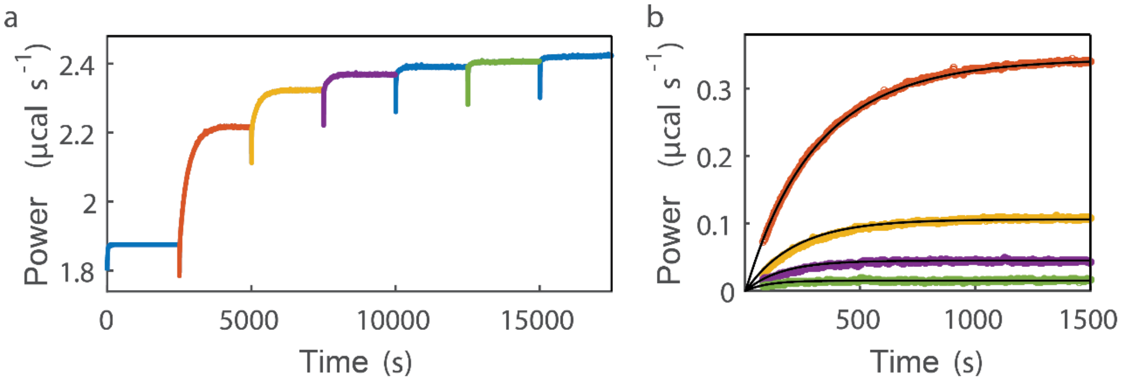

Supplementary Figure 13. Kinetics of inhibition experiment using compound 3. a) Baseline corrected data. 7 injections (1st injection 0.3 µL over 0.6 s, subsequent 6 injections 5 µL over 8 s) of compound **3** (462 nM in syringe) into cell containing POP and TRH (7.64 nM and 25.2 mM) at 30°C in buffer described above using a Malvern ITC-200. b) Overlay of injections 2 (open orange circles), 3 (open yellow circles), 4 (open purple circles) and 6 (open green circles) from baseline corrected ITC kinetics of inhibition data. Fit using the kinetics of inhibition model described above (black lines). Only 4 curves are shown for clarity only, all injections except the first was used in the fitting routine. Only fitted portion of the experimental curves are shown.

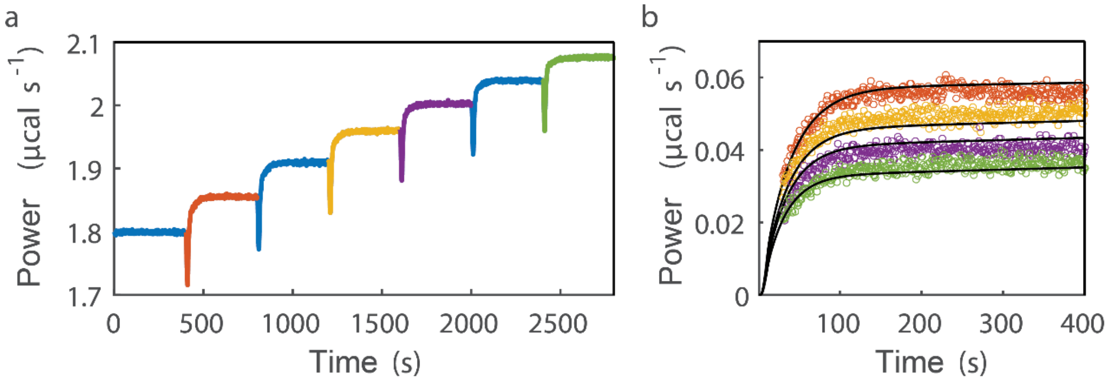

Supplementary Figure 14. Kinetics of inhibition experiment using compound 4. a) Baseline corrected data. 7 injections (1st injection 0.3 µL over 0.6 s, subsequent 6 injections 4 µL over 8 s) of compound **4** (500 nM in syringe) into cell containing POP and TRH (14.8 nM and 20 mM) at 30°C in buffer described above using a Malvern ITC-200. b) Overlay of injections 2 (open orange circles), 4 (open yellow circles), 5 (open purple circles) and 7 (open green circles) from baseline corrected ITC kinetics of inhibition data. Fit using the kinetics of inhibition model described above (black lines). Only 4 curves are shown for clarity only, all injections except the first was used in the fitting. Only fitted portion of the experimental curves are shown.

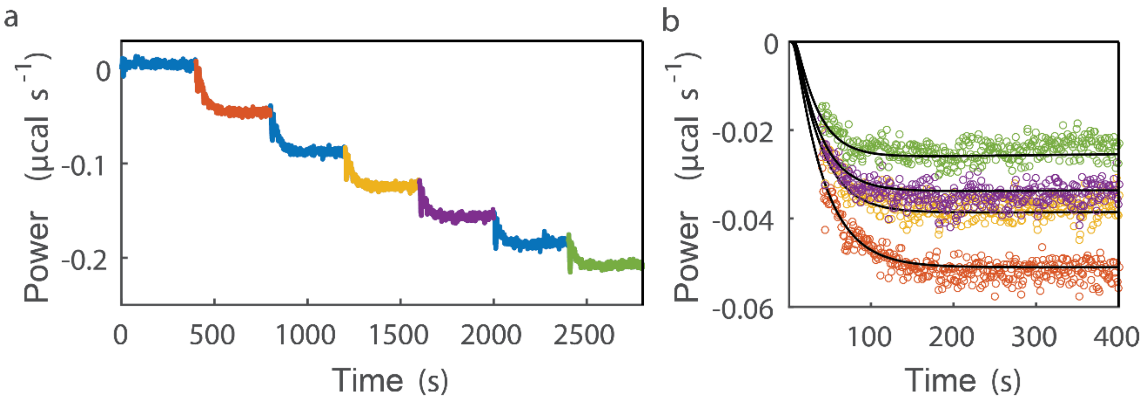

Supplementary Figure 15. Kinetics of initiation experiment using compound 4. a) Baseline corrected data, 7 injections (1st injection 0.3 µL over 0.6 s, subsequent 6 injections 2.9 µL over 5.8 s) of pre-incubated POP and compound **4** (88.1 nM and 500 nM respectively in syringe) into the cell containing TRH (6.9 mM) at 30°C in buffer described above using a Malvern ITC-200. b) Overlay of injections 2 (open orange circles), 4 (open yellow circles), 5 (open purple circles) and 7 (open green circles) from baseline corrected ITC kinetics of inhibition data. Fit using the kinetics of inhibition model described above (black lines). Only 4 curves are shown for clarity only, all injections except the first was used in the fitting. Only fitted portion of the experimental curves are shown.

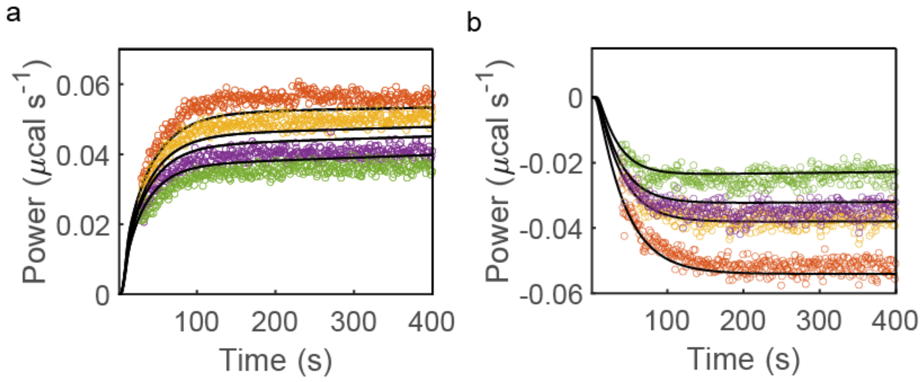

Supplementary Figure 16. Global fit for compound 4. Results of global fit between (a) inhibition experiment and (b) initiation experiment for compound **4**.

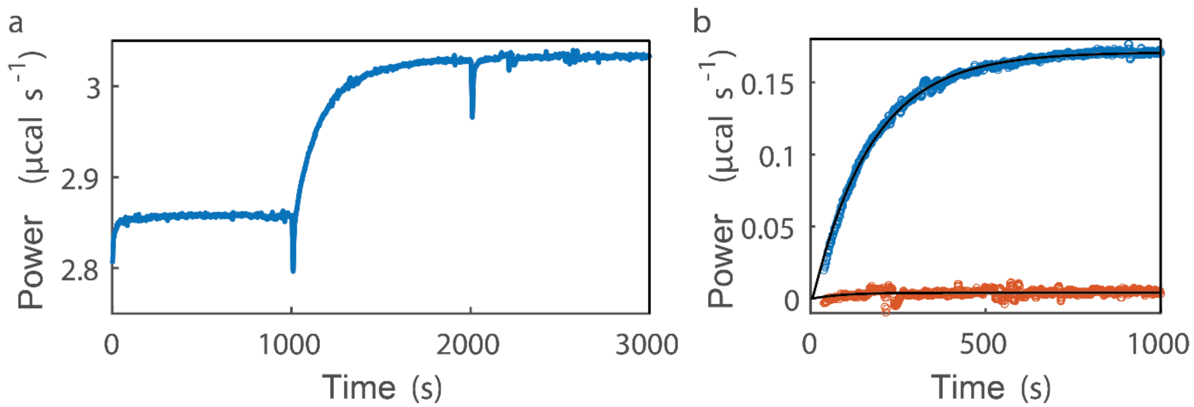

Supplementary Figure 17. Kinetics of inhibition experiment using compound 5. a) Baseline corrected data. 3 injections (1st injection 0.1 µL over 0.2 s, subsequent 2 injections 3.5 µL over 7 s) of compound **5** (11.7 µM in syringe) into cell containing POP and TRH (3.1 nM and 13.8 mM) at 30°C in buffer described above using a Malvern ITC-200. b) Overlay of injections 2 (open blue circles), 3 (open orange circles) from baseline corrected ITC kinetics of inhibition data. Fit using the kinetics of inhibition model described above (black lines). All injections except the first was used in the fitting routine. Only fitted portion of the curves are shown.

UV-Vis and NMR Spectroscopy experiments

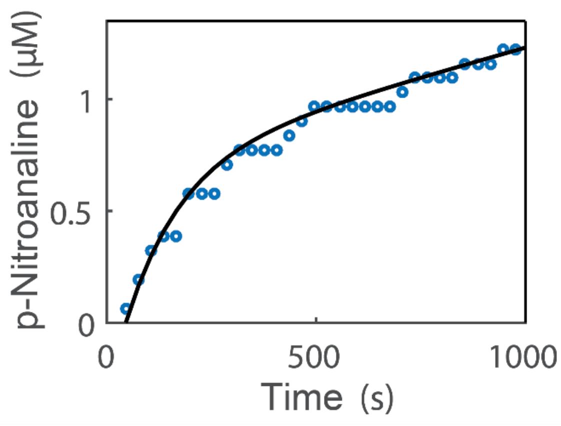

Supplementary Figure 18. UV-Vis inhibition experiment using compound 1. POP and colorimetric substrate ZGP-pNA (1 nM and 80 µM respectively) were spiked with compound **1** (final concentration of 105 nM). The absorbance at 405 nm was monitored as a function of time which was converted to a concentration of p-Nitroanaline (open blue circles). The data was fit using inhibition scripts described above (black line).

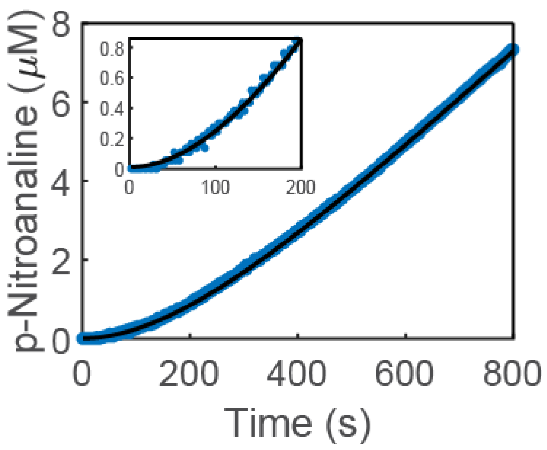

Supplementary Figure 19. UV-Vis initiation experiments using compound 1. POP and compound **1** at an original concentration of (277 nM and 1.5 μM respectively) are diluted 100-fold into buffer containing ZGP-pNA (80 μM). The intensity of the peak at 405 nm was monitored with time and converted to a concentration of p-Nitroanaline (open blue circles). The data was fit using initiation scripts similar to those described above (black line).

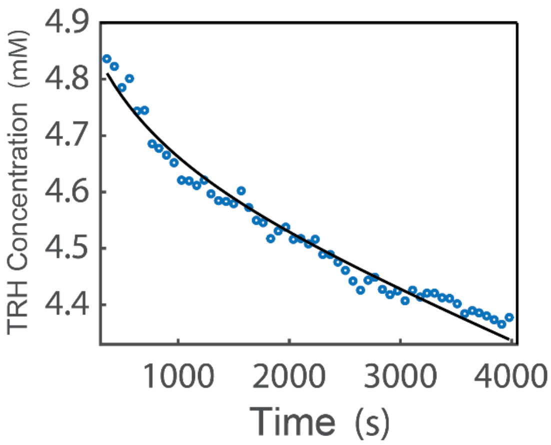

Supplementary Figure 20. NMR inhibition experiment using compound 5. POP and TRH (50 nM and 5 mM respectively) were spiked with compound **5** (final concentration of 50 nM). The peak corresponding to TRH at 8 ppm monitored as a function of time which was converted to a concentration of TRH (open blue circles). The data were fit using inhibition scripts described above (black line).

Statistical analysis of errors. Errors were calculated using residual sum of squares contour plots. This was accomplished by performing a grid search for each pair of fitted parameters for each fit and calculating the RSS at each point. The confidence level (*CL*) at each RSS value was calculated as[15]

$$CL_{i,j} = F_{CDF}\left(\left(\frac{RSS_{i,j}}{RSS_{min}} - 1\right) * \left(\frac{Dof}{M}\right), M, Dof\right) \quad (46)$$

where F_{cdf} is the F distribution cumulative density function and RSS_{min} is the residual sum of squares at the minimum found via fitting. $RSS_{i,j}$ is the RSS at the point i,j for each the i/jth pair of fitted parameters. Dof is the degrees of freedom of the fit and M is the total number of fitted parameters. The resulting %CL contour plots are shown below with the 95%CL contour as a black dotted line, for selected pairs of parameters. Errors are shown in **Table 1** of the main text and **Supplementary Table 3**.

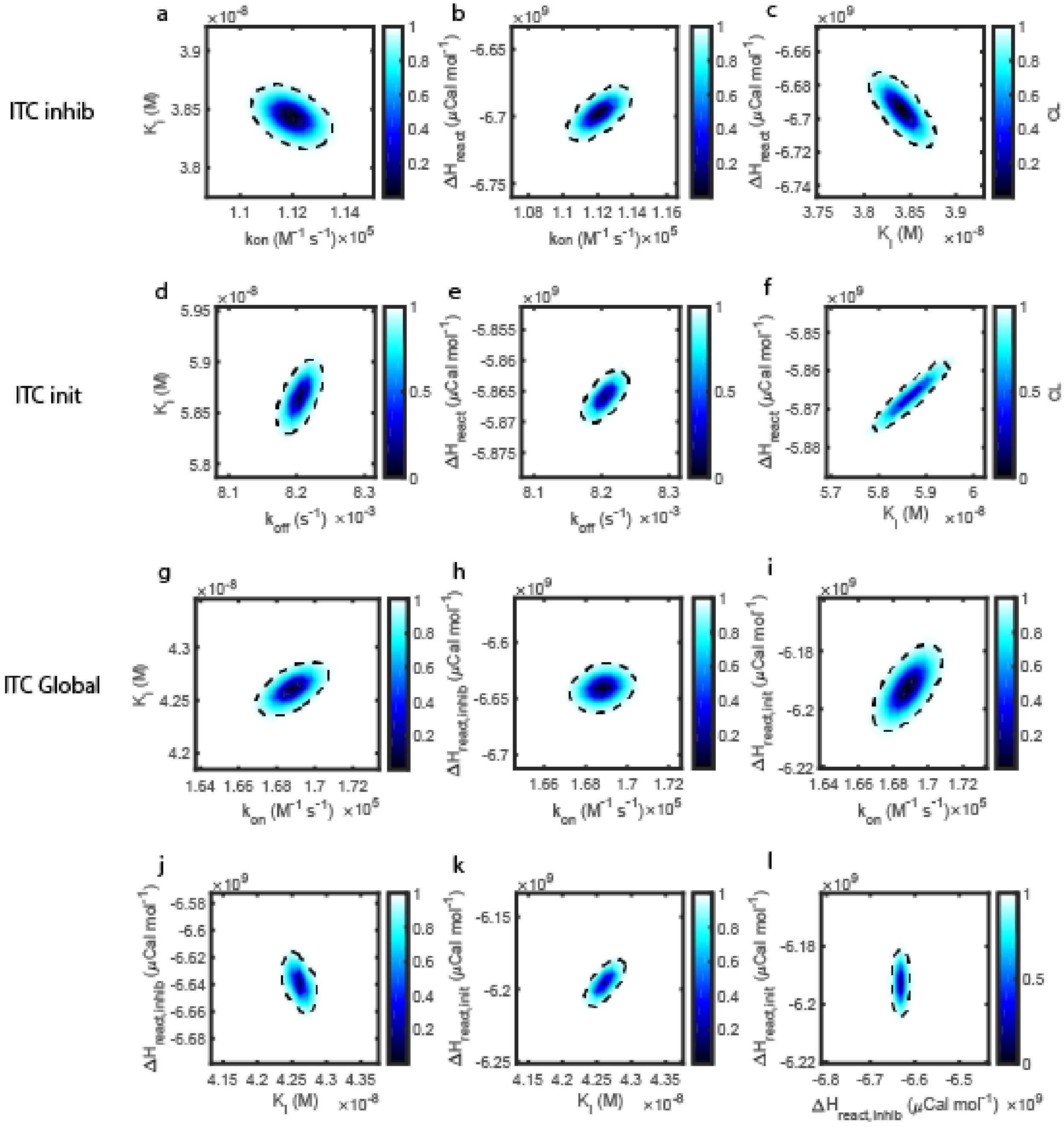

Supplementary Figure 21. Confidence level contour plots for ITC experiments using compound 1. Error surfaces for each 0pair of fitted parameters for a)-c) kinetics of inhibition experiment, d)-f) kinetics of initiation experiment and, g)-l) global fit between inhibition and initiation experiment. Dotted line highlights the 95%CL. The largest error value at the 95%CL is reported for each parameter.

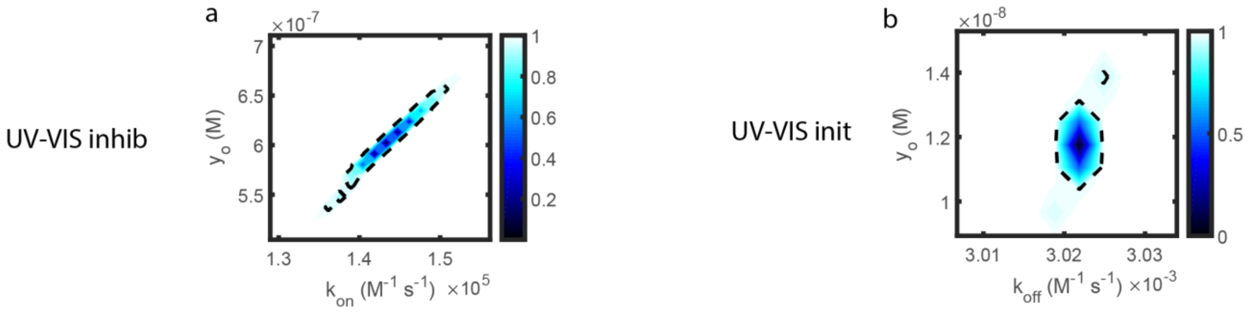

Supplementary Figure 22. Confidence level contour plots for UV-VIS experiments using compound 1. Error surfaces for pairs of fitted parameters for a) inhibition experiment, b) initiation experiment. Dotted line highlights the 95%CL. The largest error value at the 95%CL is reported for each parameter.

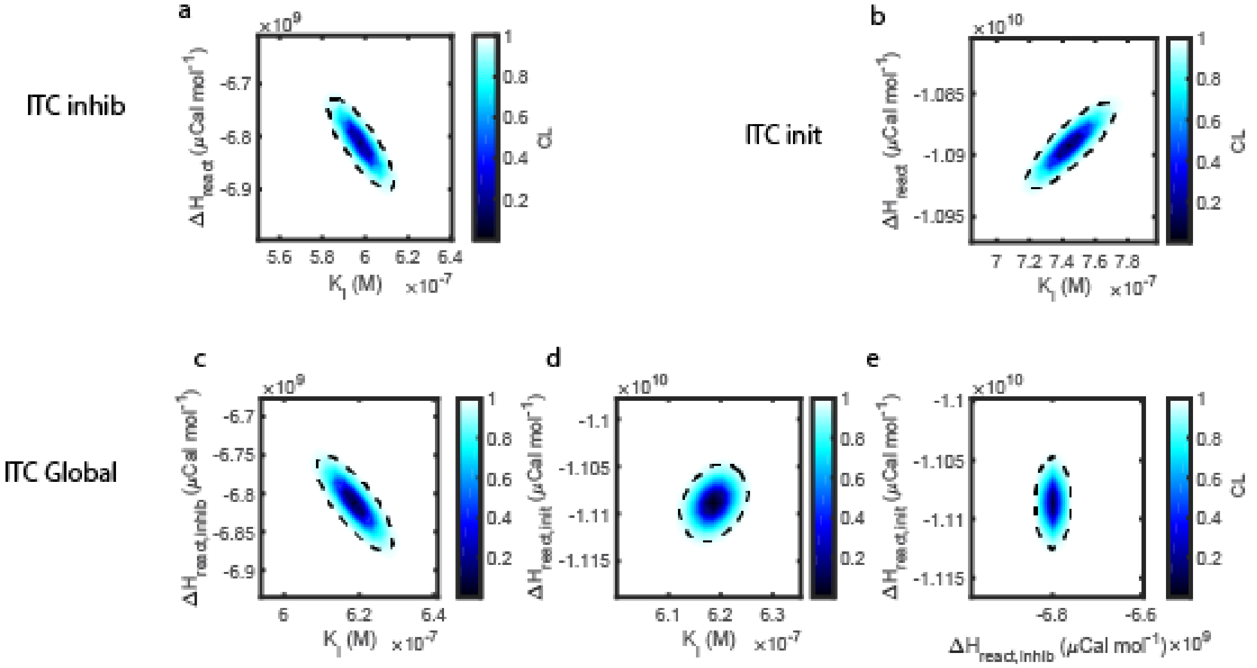

Supplementary Figure 23. Confidence level contour plots for ITC experiments using compound 2. Error surfaces for each pair of fitted parameters for a) kinetics of inhibition experiment, b) kinetics of initiation experiment and, c)-e) global fit between inhibition and initiation experiment. Dotted line highlights the 95%CL. The largest error value at the 95%CL is reported for each parameter.

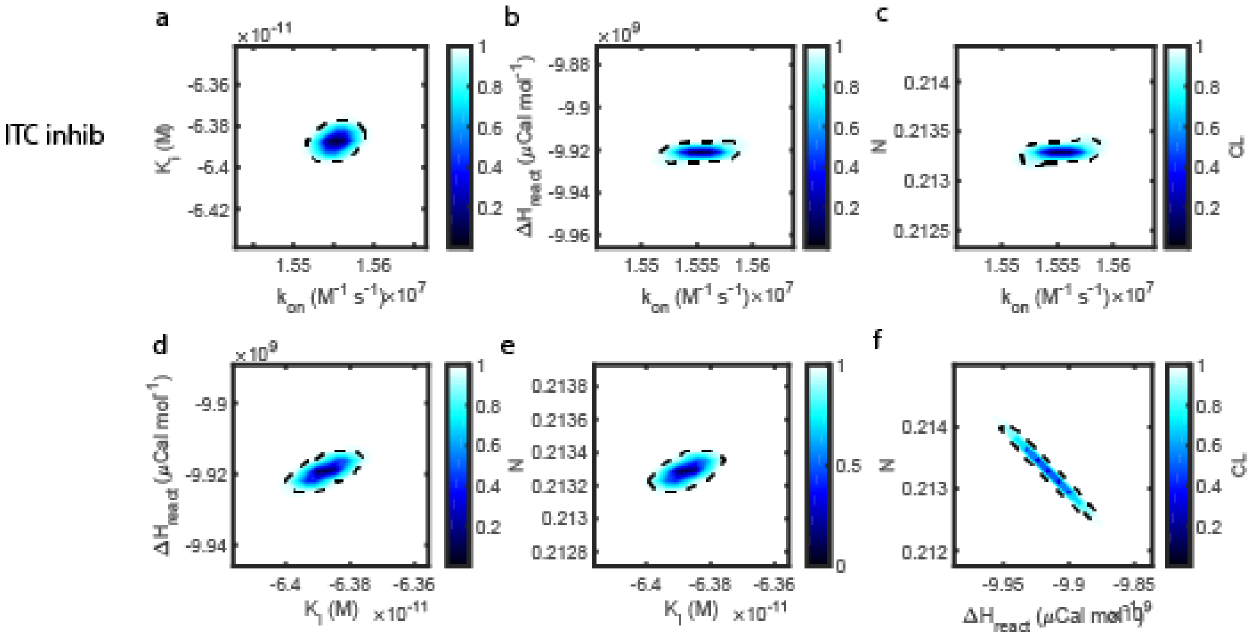

Supplementary Figure 24. Confidence level contour plots for ITC experiments using compound 3. Error surfaces for each pair of fitted parameters for a)-f) kinetics of inhibition experiment. Dotted line highlights the 95%CL. The largest error value at the 95%CL is reported for each parameter.

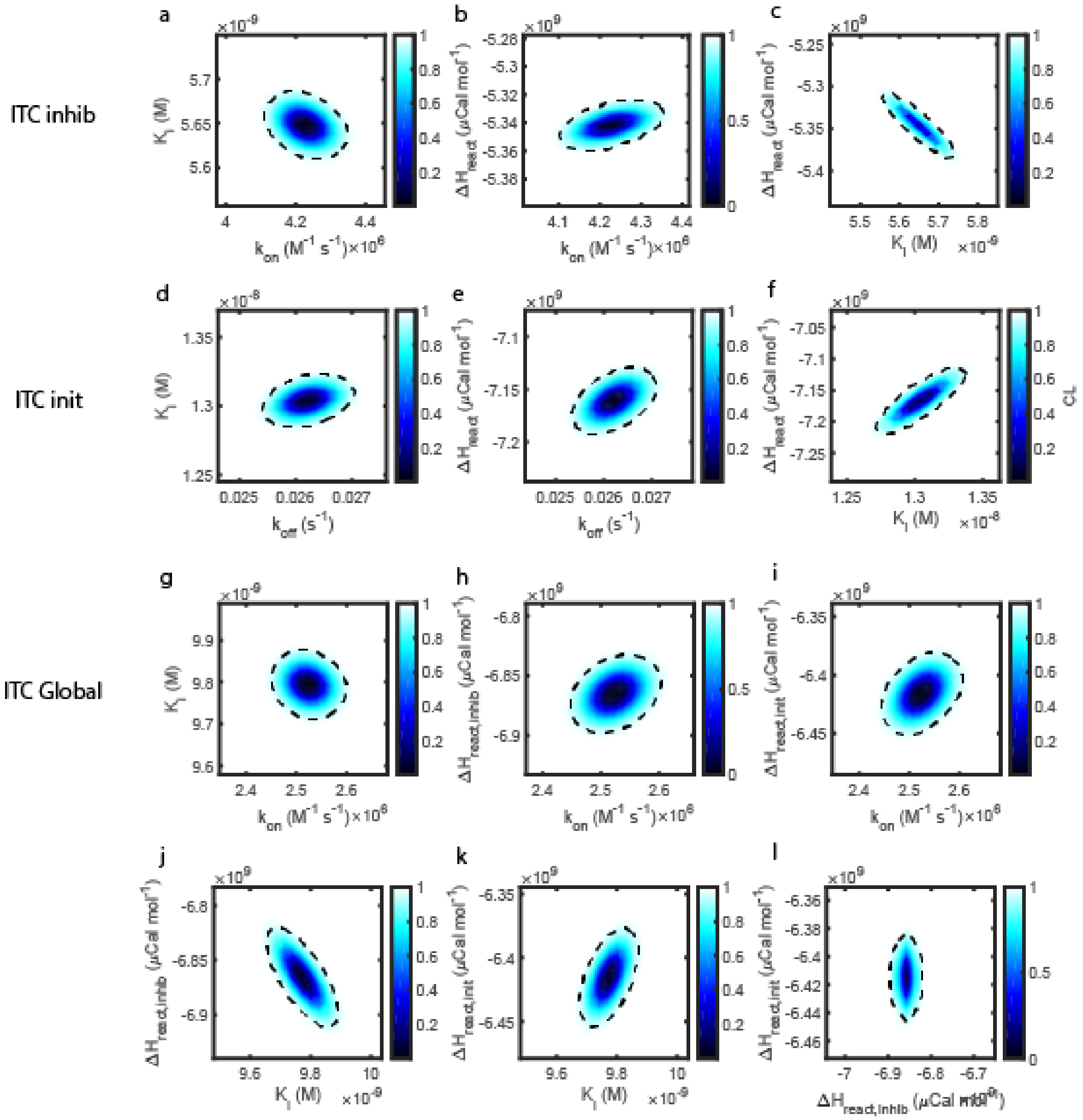

Supplementary Figure 25. Confidence level contour plots for ITC experiments using compound 4. Error surfaces for each pair of fitted parameters for a)-c) kinetics of inhibition experiment, d)-f) kinetics of initiation experiment and, g)-l) global fit between inhibition and initiation experiment. Dotted lines highlight the 95%CL. The largest error value at the 95%CL is reported for each parameter.

ITC inhib

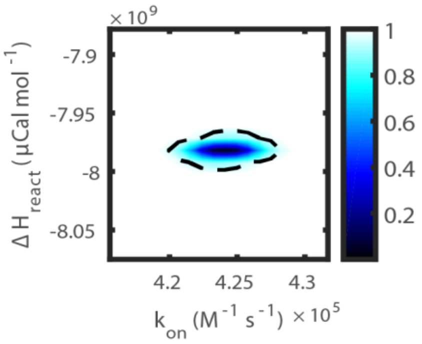

Supplementary Figure 26. Confidence level contour plots for ITC experiments using compound 5. Error surfaces for each pair of fitted parameters for kinetics of inhibition experiment. Dotted line highlights the 95%CL. The largest error value at the 95%CL is reported for each parameter.

NMR inhib

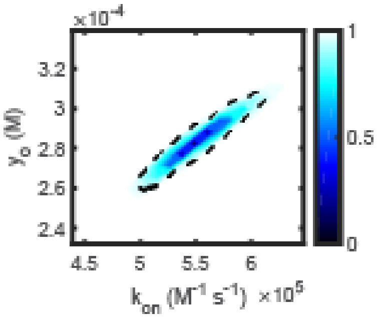

Supplementary Figure 27. Confidence level contour plots for NMR experiments using compound 5. Error surfaces for each pair of fitted parameters for kinetics of inhibition experiment. Dotted line highlights the 95%CL. The largest error value at the 95%CL is reported for each parameter.

Supplementary Table 3. Kinetic and thermodynamic parameters.

Cpd	Experiment	$\mathbf{k_{on}}$ ×10^5 M^{-1} s^{-1}	$\mathbf{k_{off}}$ ×10^{-4} s^{-1}	$\mathbf{K_i}$ nM	[b]$\mathbf{\Delta H_{cat}}$ kcal mol^{-1}
1	ITC Inhib.	1.12 ± 0.02	43.1[a] ± 0.9	38.4 ± 0.4	-6.69 ± 0.02
	ITC Init.	1.21[a] ± 0.01	82.1 ± 0.4	58.7 ± 0.5	-5.86 ± 0.01
	ITC Global	1.68 ± 0.02	72 ± 1	42.6 ± 0.4	-6.32 ± 0.02, -6.18 ± 0.01
	UV-VIS	1.43 ± 0.02	31.4 ± 0.3	947[a] ± 2	
2	ITC Inhib.	--	--	597 ± 28	-6.79 ± 0.03
	ITC Init.	--	--	744 ± 16	-10.88 ± 0.06
	ITC Global	--	--	618 ± 10	-6.79 ± 0.05, -11.07 ± 0.03
3	ITC Inhib.	155.5 ± 0.4	9[a] ± 1.5	0.063 ± 0.01	-9.91 ± 0.03
4	ITC Inhib.	44 ± 1	249[a] ± 7	5.6 ± 0.1	-5.33 ± 0.03
	ITC Init.	20[a] ± 1	261 ± 9	13.1 ± 0.4	-7.19 ± 0.04
	ITC Global	25.2 ± 0.2	250 ± 8	9.72 ± 0.3	-6.85 ± 0.03 , -6.41 ± 0.02
5	ITC Inhib.	4.18 ± 0.04	< 1.05	< 2.5	-7.97 ± 0.01
	NMR	5.4 ± 0.7	--	--	
	Lit[14]	0.7	3	0.5	

[a]calculated using $K_i = k_{off}/k_{on}$

[b]ΔH_{cat} are floated in order account for uncertainties in enzyme concentration as the magnitude of the heat flow is directly proportional to [Eo]ΔH_{cat}. Since the inhibition and initiation experiments are run separately, different ΔH_{cat} correction terms can be obtained for the two experiments.

SUPPLEMENTARY NOTE 2 - GENERAL CONSIDERATIONS

1 - Introduction

The following is intended to assist potential users with experimental design and data interpretation and also explores the ranges of applicability for the techniques. Guidelines are given for selecting enzyme, substrate, and inhibitor concentrations, largely based on the Malvern ITC-200 instrument and the physical parameters of the POP/inhibitor system studied here (ΔH_{cat} , K_m, $k_{cat,}$ k_{on} and K_i). We expect these guidelines to apply generally but the precise values will depend to some extent on the system under investigation and the instrument used. For POP, we estimate the upper and lower limits for measurable k_{on}, k_{off}, values to be (8×10^6 and 3×10^3 M^{-1} s^{-1}) and (0.1 and 5×10^{-4} s^{-1}) respectively. The lower limits for the K_i values are estimated to be 30 pM for inhibition experiments and 1 pM for initiation experiments respectively.

2 - Instrument parameters

2.1 - Pre injection delay

For optimal results, it is advisable to allow the baseline to reach a steady power level prior to starting the experiment. We used long pre-injection delays of 1000-2000s in order to ensure baseline stability.

2.2 - Injection volume

It is advisable to follow the minimum and maximum injection volume guidelines provided by the instrument manufacturer. Injections that are too large can produce artifacts due to temperature differences between the injectant and the cell contents.[12] The largest injection size used in this study using the Malvern ITC-200 is 13 µL in the inhibition experiments with compound **1**. Injections that are too small

can lead to large relative errors in volume, partly due to diffusion of the injectant from the tip of the syringe between injections. We used a minimum injection size of 2 µL.

2.3 – Spacing between injections

We recommend inter-injection spacing long enough for the signal to completely stabilize at each new plateau (according to the kinetics of the inhibitor) and to accurately evaluate the new baseline (and additional 200-300s) after each injection.

3 - Kinetic limitations

3.1 - Upper kinetic rate limits

The upper and lower limits for rate constants are largely determined by the magnitude of the change in power signal, the instrument noise level, and the response time of the instrument. In order to estimate the minimum signal to noise ratio (SNR_{min}) required to extract reliable rate constants from single exponentially-decaying data, a series of datasets were generated according to $S(t) = SNR \times \exp\{-k_{react}t\} + \sigma(t)$, sampled in 1-second intervals, with $k_{react} = 0.1\ s^{-1}$, SNR varying from 11 to 3.9, and values of $\sigma(t)$ drawn randomly from a Gaussian distribution with a mean of 0 and standard deviation of 1 (1000 curves for each SNR). Each curve was fit individually to $S_{fit}(t) = A \times \exp\{-k_{react}t\}$ while floating the values of A and k_{react}. The relative error of k_{react} at each value of SNR was calculated as the standard deviation divided by the true value of the parameter ($0.1\ s^{-1}$) (**Supplementary Fig. 28**). We find that reliable rate constants can be extracted when the magnitude is at least 4-fold greater than the baseline noise, i.e. $SNR_{min} = 4$, which is consistent with experimental data (**Supplementary Fig. 29**).

For estimating the upper kinetic limit, we take the short-timescale baseline noise to be $Q_{RMS} = 0.0017\ \mu cal\ s^{-1}$. Assuming that the signal decays exponentially from the end of the injection (τ_{inj}) to the beginning of

the analysis period (τ_{blank}, see above), i.e. for approximately $3\times\tau_r$, where τ_r is the instrument response time, the maximum extractable rate is given by

$$k_{max} = (3\tau_r)^{-1} ln\left\{\frac{\Delta Q_{inj}}{SNR_{min}\times Q_{RMS}}\right\} \tag{47}$$

The average ΔQ_{inj} over all compounds is ~0.1 μcal s^{-1} in this study, giving a rough upper limit of 0.09 s^{-1}, which corresponds to the maximum extractable dissociation rate. To estimate the maximum extractable association rate, we assume an inhibitor concentration of 11 nM (used for compound **3**) giving an approximate maximum of 8.1×10^6 M^{-1} s^{-1} for similar systems.

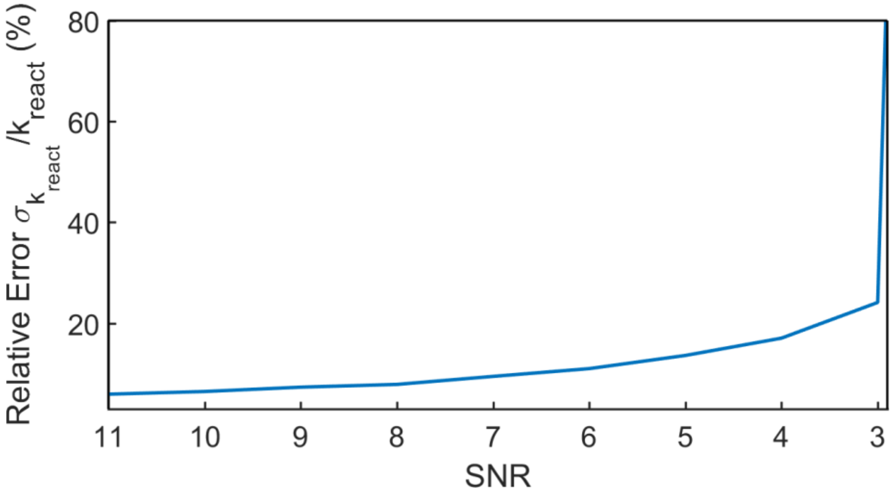

Supplementary Figure 28. Monte Carlo simulations of first order kinetics experiments. Relative error of the k_{react} vs the signal to noise ratio (SNR).

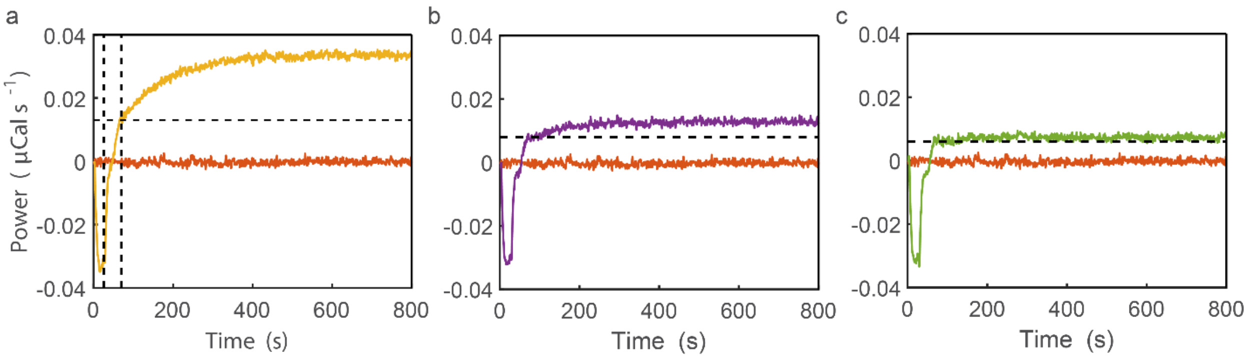

Supplementary Figure 29. Experimental confirmation of SNR_{min}. a) Second, b) third and c) fourth injection of kinetics of inhibition experiment using compound **1**. Vertical dashed lines were placed at $\boldsymbol{\tau}_{inj}$ and $\boldsymbol{\tau}_{blank}$ in a). Horizontal dashed line was placed where the experimental curve passes $\boldsymbol{\tau}_{blank}$ for all injections. The ΔQ_{inj} calculated as the difference between the maximum signal and the horizontal dashed line for a) is 0.029 µcal s^{-1} , b) is 0.008 µcal s^{-1} and c) is 0.0015 µcal s^{-1} making the SNR ~17, 4.7 and 0.9 respectively. When individually fit the curves from panel a) and b) can be used to extract precise kinetic rates while c) cannot. This is consistent with the data form the simulations above.

3.2 - Lower kinetic rate limits

The lower limit for measurable kinetics is largely determined by the rate of baseline drift and the magnitude of the change in power signal. In order to estimate the baseline drift, we performed several experiments consisting of long baseline measurements with no injections. We then calculated the root-mean-square deviation in power as a function of time according to

$$Q_{RMS}(\tau) = \sqrt{\langle \left(Q(t) - Q(t+\tau)\right)^2 \rangle} \tag{48}$$

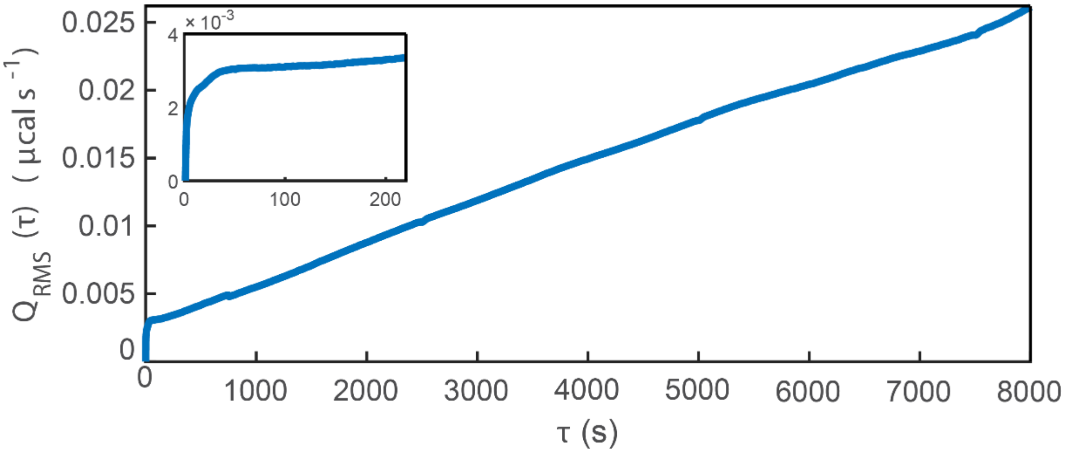

Supplementary Figure 30. Q_{RMS} vs. τ. 5 x 9000s experiments with pre injection delays of 2000s with buffer described in the materials and methods section were run and analysed individually using **equation 48** then averaged together. Only the first 8000s is shown as the Q_{RMS} values become increasingly poorly defined for large **τ**. The inset shows first 220s.

Q_{RMS} shows a rapid rise as short times due to random noise and fast-timescale fluctuations, followed by a more gradual increase corresponding to long-timescale drift, reaching 0.025 µcal s^{-1} after 8000s. The kinetic ITC signals have 95% reached their new baselines after a length of time given by 4/k. We can thus use a plot of $Q_{RMS}(\tau)$ and the magnitude of the signal, ΔQ_{total}, to estimate the longest accessible rate constants by finding the value of k_{min} which satisfies

$$Q_{RMS}\left(\tau = \frac{4}{k_{min}}\right) \times SNR_{min} = \Delta Q_{inj} \qquad (49)$$

For the baseline measurements made here, Q_{RMS} appears to increases linearly with τ, the Q_{RMS} plot shown in **Supplementary Fig. 30** fits well to a line starting at τ = 50 s yielding a slope and intercept of 2.89×10^{-6} and 2.99×10^{-3} respectively. Using an average ΔQ_{inj} of 0.1 µcal s^{-1} and Q_{RMS} = 0.0017 µcal s^{-1}, this gives an approximate k_{min} of 5.26×10^{-4} s^{-1}, which corresponds to the minimum value of k_{off} and, for an inhibitor concentration of 193 nM (inhibition experiments with compound **5**), this gives a minimum association rate of 2.73×10^{3} M^{-1} s^{-1}.

4 - Kinetics of inhibition experiments

4.1 - Measuring thermodynamic and kinetic properties for an enzyme-substrate pair

It is recommended that the ΔH_{cat}, k_{cat} and K_m values are measured prior to designing experiments, for instance by the procedure of Gomez et al[13] (see **Prolyl oligopeptidase enthalpy of catalysis and Michaelis-Menten parameters**).

4.2 - Enzyme and substrate concentrations

4.2.1 - Maximizing signal in inhibition experiments

The sensitivity of the kinetics experiments is optimized by maximizing the changes in heat flow. In the case of inhibition experiments, this is achieved by maximizing the total heat flow due to catalysis

$$\Delta Q_{total} = \Delta H_{cat} * V_{cell} \frac{k_{cat} * E_{o,cell} * S}{K_m + S} \tag{50}$$

Where ΔH_{cat} is the enthalpy of catalysis, V_{cell} is the volume of the cell, k_{cat} and K_m are the catalytic rate and Michaelis-Menten constant respectively, S is the total amount of substrate in the cell and $E_{o,cell}$ is the total amount of enzyme in the cell. This is particularly true as the signal is split into multiple ΔQ_{inj} increments. ΔQ_{total} can be optimized by maximizing the amount of enzyme and using saturating substrate concentrations. As well, when several different substrates are available, the ones with the largest $|\Delta H_{cat}|$ values are preferable. Under the experimental conditions used in this study we calculate the baseline noise level to be 0.0017 μcal s^{-1}. In the experiments presented here the smallest SNR was obtained in inhibition experiments with compound **2** (see **Supplementary Fig. 10**). Here the total $\Delta Q_{total} \approx 0.2$ µcal s^{-1}, SNR ≈ 67. Split between 7 injections, this gives an average SNR per injection of just under 10.

4.2.2 – Steady rate enzyme kinetics in inhibition experiments

In order to achieve a steady baseline signal, substrate concentrations must remain either fully saturating or essentially constant at a sub-saturating level. Significant depletion of substrate over the course of an

experiment it will lead to large baseline drift, making the results difficult to interpret. It is therefore important to ensure that

$$S \gg \frac{k_{cat} * \langle E_{o,cell} \rangle * S}{K_m + S}\ \tau_{exp} \tag{51}$$

Where τ_{exp} is the total time of the experiment and angled brackets indicate the average free enzyme concentration over the course of the experiment. All of the experiments performed in this study were prepared such that the left hand side of **equation 51** is at 5-fold or more greater than the right hand side.

4.2.3 – c-value and its implications for experimental design

In standard ITC assays, the c-value is calculated as K_D/[M], where K_D is the equilibrium dissociation constant and [M] is the concentration of macromolecule in the cell. When designing enzyme inhibition kinetics studies, a modified c-value can be calculated as $IC_{50}/[E_o]$, as the amount of inhibitor needed to achieve 50% inhibition is not equal to the K_i, in general. For competitive inhibitors $IC_{50} = K_i$ x $(1+ S/K_m)$, for uncompetitive inhibitors $IC_{50} = K_i$ x $(1+ S/K_m)^{-1}$, and for noncompetitive inhibitors, $IC_{50}=K_i$. At very high c-values (greater than about 5.8), it becomes difficult to extract reliable K_i values (see **4.4.2 – Monte Carlo simulations for the lower K_i limit**). The c-value can be lowered by decreasing the concentration of enzyme and by altering concentration of substrate in the case of competitive and uncompetitive inhibitors.

4.3 – Inhibitor concentration

4.3.1 – Tailoring inhibition kinetics

The majority of the inhibition experiments performed in this study have a large excess of inhibitor compared to enzyme present in the cell. This means that the change in inhibitor concentration will be negligible throughout and during an injection making it possible to express the rate of inhibition in terms of a pseudo first order rate constant ($k_{on}' = k_{on}$ x $[I]_{cell}$) with units of s^{-1} where $[I]_{cell}$ is the concentration of

inhibitor in the cell following each injection. It is therefore possible to tune the effective kinetics in the inhibition experiments by altering the concentration of inhibitor.

4.3.2 – Decreasing inhibitor concentration

For compounds with very rapid binding kinetics it is possible to decrease the inhibitor concentration such that k_{on}' is smaller than the upper kinetic rate limit (see **3.1 - Upper kinetic rate limits**). One difficulty with decreasing the inhibitor concentration is that it leads to a smaller change in the fractional inhibition of the enzyme and therefore a smaller signal (ΔQ_{inj}). For example, we were unable to measure the kinetics of inhibition for compound **2** due to its large K_i. High concentrations of inhibitor were required to achieve measurable ΔQ_{inj} signals, such that k_{on}' was far above the upper kinetics rate limit. In contrast, compound **4** bound much more tightly allowing much smaller concentrations of inhibitor to be used, such that k_{on}' was measurable.

4.3.3 – Increasing inhibitor concentration

In principle it is always possible to increase the inhibitor concentration such that k_{on}' is larger than the lower rate limit (see **3.2 - Lower rate kinetic limits**). In cases where both k_{on} and K_i are very low this may lead to K_i becoming experimentally inaccessible. Raising the concentration of a slow binding inhibitor in order to increase k_{on}' can lead to the enzyme becoming saturated with inhibitor after the first injection, as seen for compound **5**.

4.4 – Limitations for the K_i

4.4.1 – Upper limit for K_i

In principle, the only factor setting the upper limit for K_i (lower limit for affinity) is the magnitude of the changes in heat flow with each injection. This can be maximized by increasing the inhibitor concentration (limited by inhibitor solubility) and enzyme concentration (taking care not to overly deplete the substrate; see **4.2 - Enzyme and substrate concentrations**). For competitive inhibitors lowering the substrate

concentration will help raise the c-value and increase the amount of binding with each injection, but will limit the amount of enzyme that can be used, due to substrate depletion during the experiment. In this study the highest K_i values we measured was for compound **2** (~650 nM). Lower affinities (larger K_i's) are measurable but not necessarily of practical interest for drug design.

4.4.2 – Monte Carlo simulations for the lower K_i limit

The lower limit for measurable K_i values largely depends on the modified c-value (**see Section 4.2.3 c-value and its implications for experimental design**). Note that the dependence on the c-value differs from ITC experiments performed in standard binding mode as these detect the heat released directly by binding whereas the kinetic assays detect binding indirectly as the change in heat released by catalysis. In order to map out the lower limit, we performed a Monte Carlo analysis in which data were simulated using POP enzymatic parameters, with baseline noise (0.0017 µcal s^{-1}), 1% error in the injection size (Malvern ITC-200 manual). Enzyme concentrations were set at 5 nM, substrate concentration at 25 mM, values of K_i varied from 10 nM to 10 pM, and inhibitor concentrations ranged 1 µM for high K_i simulations to 50nM for low K_i simulations. In order save computational time a simplified (fast-limit) version of the inhibition model was used to simulate and fit data in which all injected inhibitor binds instantaneously. 1000 synthetic datasets were generated for each value of K_i. Each data set was fit individually and the relative error in K_i was calculated as the standard deviation divided by the value of the parameter (**Supplementary Fig. 31**).

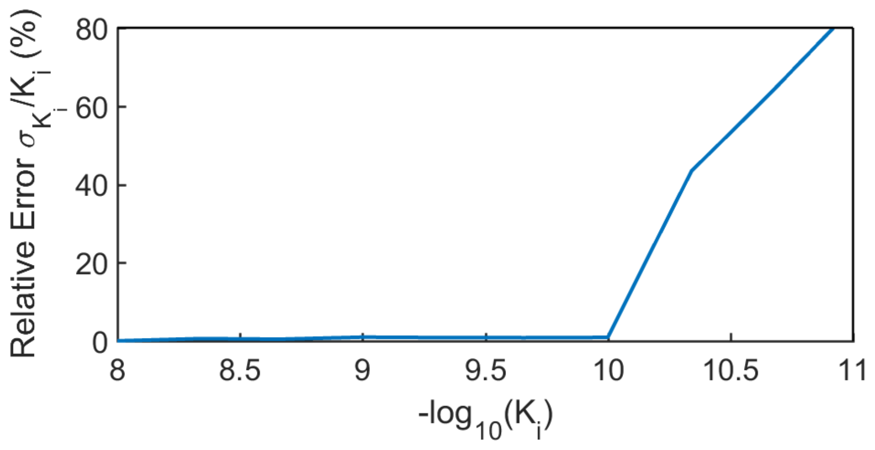

Supplementary Figure 31. Monte Carlo simulations of inhibition experiments. Relative error of the K_i vs the $-\log_{10}(K_i)$.

Robust K_i values are obtained down to a value of about ~0.1 nM at which point the relative error begins to increase rapidly. At $-\log_{10}(10.5)$ (32pM) the relative error is approximately 50% making this an approximate lower limit measuring K_i with the kinetics of inhibition experiment Note that at the concentrations used in the simulation, this corresponds to a c-value of 5.8. Our lowest experimental K_i was 60 pM for compound **3**. The estimated relative experimental error was approximately 15%, which is consistent with the errors calculated in these simulations. When measuring high affinities, it can be helpful to reduce the enzyme concentration as much as possible and to adjust the substrate concentration to increase the IC_{50}. (see **4.2 – Enzyme and substrate concentrations**).

4.5 – Setting up inhibition experiments

1. Clean the ITC thoroughly as per manufacturer's instructions.
2. Pre-equilibrate ITC to the experimental temperature to help the baseline reach equilibrium more rapidly once the experiment is initiated.
3. Select experimental parameters – all the experimental parameters (injection size, length etc. see **2- Instrument parameters**) should be chosen at this time so that the experiment is ready to initialize.
4. Make enzyme and substrate solutions separately to ensure that substrate is not consumed prior to the experiment being initiated.
5. Load syringe with inhibitor – the inhibitor should be dissolved in a buffer that most closely matches the buffer in the cell in order to minimize dilution artifacts.
6. Mix enzyme and substrate to initiate catalysis.

7. Load the enzyme-substrate mixture into reaction cell.
8. Initiate experiment

5 - Kinetics of initiation experiments

5.1 - Measuring thermodynamic and kinetic properties for an enzyme-substrate pair

See section **4.1 - Measuring thermodynamic and kinetic properties for an enzyme-substrate pair**

5.2 - Enzyme and substrate concentrations

5.2.1 – Maximizing signal in initiation experiments

As for the kinetics of inhibition experiment, the total signal for a kinetics of initiation ITC experiment is maximized by using the largest concentrations of enzyme and substrate available. The magnitude of the signal can be calculated as above using **equation 50** (see **4.1.1 - Maximizing signal in inhibition experiments**), calculating $E_{o,cell}$ as $E_{o,syr*}(1\text{-}\exp(-v_{inj,total}/V_{cell}))$ where $v_{inj,total}$ is the total volume injected throughout the experiment.

5.2.2 - Steady rate enzyme kinetics in initiation experiments

Similarly to the kinetics of inhibition experiments, substrate concentrations must remain constant or saturating in kinetics of initiation experiments in order to keep a steady rate of catalysis (see **4.2.2 – Steady rate enzyme kinetics in inhibition experiments**). **Equation 51** can once again be used, calculating $E_{o,cell}$ as $E_{o,syr*}\exp(-v_{inj,total}/V_{cell})$. All of the experiments performed in this study were prepared such that the left hand side of **equation 51** is at a minimum over 5 times the right hand side. This introduces an upper limit for the enzyme concentration and a lower limit for the substrate concentration initiation experiments.

5.2.3 - Substrate concentration

In principle having a large substrate concentration is always advantageous in kinetics of initiation experiments, this is because it maximizes signal and ensures steady rate kinetics (see **5.2.1** and **5.2.2**

above). In the case of competitive inhibitors, high concentrations of substrate also favour enzyme-inhibitor dissociation (see **5.4 – Optimizing signal due to dissociation**).

5.3 – Limitations for the K_i

5.3.1 – Upper limit for K_i

Similarly to the kinetics of inhibition experiments, the main factor setting the upper limit for K_i (lower limit for affinity) is the magnitude of the changes in heat flow with each injection. This can be maximized by increasing the concentrations of inhibitor and enzyme in the syringe, taking care not to overly deplete the substrate. For competitive inhibitors increasing the substrate concentration will help lower the c-value and increase the amount of dissociation that occurs with each injection (see **5.4 – Optimizing signal due to dissociation** below). In this study the highest K_i value we measured was for compound **2** (675 ≈ nM). Lower affinities (larger K_i's) are measurable but not necessarily of practical interest for drug design.

5.3.2 – Monte Carlo simulations for the lower K_i limit

In order to estimate the lowest K_i values measurable by this technique, we performed a Monte Carlo analysis in which data were simulated using POP enzymatic parameters, with baseline noise (0.0017 µcal s^{-1}) and 1% error in the size in the injection (Malvern ITC-200 manual). Simulated values of K_i varied from 10 nM to 0.1 pM with enzyme concentrations ranging from 800 nM for the high K_i to 400 nM for the low K_i. Inhibitor concentrations were set to $1.2\times[E_o]_{syr}$ and the substrate to 20 mM. In order save computational time a simplified (fast-limit) version of the initiation model was used to simulate and fit data in which all injected EI complex dissociates instantaneously. 1000 synthetic datasets were generated for each value of K_i. Each data set was fit individually and the relative error in K_i was calculated as the standard deviation divided by the value of the parameter (**Supplementary Fig. 32**).

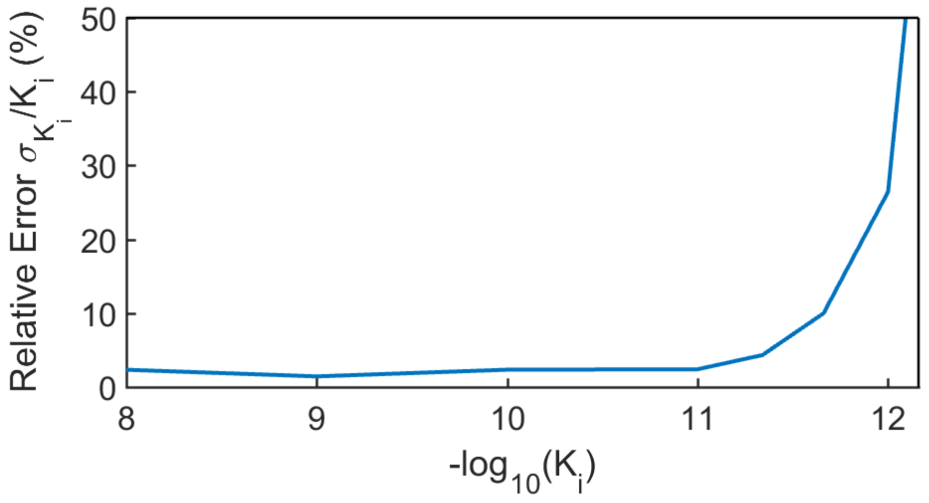

Supplementary Figure 32. Monte Carlo simulations of initiation experiments. Relative error of the K_i vs the $-\log_{10}(K_i)$.

K_i values are accurately reproduced down to a value of about 0.01 nM, at which point the relative error begins to increase sharply. At ~$-\log_{10}(12)$ (1 pM) the relative error is approximately 30% making this an approximate lower limit for extractable K_i's using the kinetics of inhibition experiment. In this study the lowest K_i we measured was 13 nM using compound **4**. This is far above the limit calculated in the simulation above. Note that the compounds that bound more tightly exhibited dissociation rates that were too slow to measure (compound **3** and **5**).

5.4 - Optimizing signal due to dissociation

5.4.1 - Dissociation of enzyme-inhibitor complex due to dilution

The magnitude of the signal in a kinetics of initiation experiment depends on the number of injected EI complexes that dissociate following an injection. Thus it is advisable to maximize the dilution factor by setting the injection volume to the minimum value recommended (see **2.2 – Injection volume**). The concentration of the inhibitor should ideally be several-fold larger than the K_i in the syringe to maximize binding and on the order of the IC_{50} or lower in the cell after the injection to ensure that dissociation

occurs. Note that dilution from 100×K_i to 10×IC_{50} does not lead to much dissociation. For competitive inhibitors, maximizing the concentration of substrate increases the IC_{50} and promotes dissociation. The concentration of enzyme in the syringe is ideally at a maximum, provided that it is lower than the concentration of the inhibitor in the syringe and does not overly deplete the substrate in the cell (see section **5.2 – Enzyme and substrate concentrations**).

5.5 - Setting up initiation experiments

1. Pre-incubate enzyme and inhibitor allowing for sufficient time for the enzyme-complex to form. In our study we pre-incubate for approximately two hours.**
2. Clean the ITC thoroughly as per manufacturer's instructions.
3. Pre-equilibrate ITC to the experimental temperature to help the baseline reach equilibrium more rapidly once the experiment is initiated.
4. Select experimental parameters – all the experimental parameters (injection size, length etc. see **2 - Instrument parameters** section) should be chosen at this time so that the experiment is ready to initialize.
5. Load syringe with the enzyme-inhibitor complex– this should be prepared in buffer that most closely matched the buffer in the cell in order to minimize dilution artifacts.
6. Add substrate solution to reaction cell.
7. Initiate experiment

** In the case of enzyme-inhibitor pairs with extremely slow association kinetics longer pre-incubation times may be necessary.

6 - Data analysis workflow

Once the experiment is complete the data is ready for analysis. Data can be analysed using the following workflow.

1. Data extraction – only the raw output provided by the instrument (power as a function of time) is needed for analysis.
2. Baseline correction – generally data must be baseline corrected (see **Baseline correction; Supplementary Fig. 1**).
    a. The last portion of each injection (see **2.3 – Spacing between injections**) should now be a flat horizontal line (see **Baseline correction; Supplementary Fig. 2**).
    b. The injections can either be analysed as a single continuous experiment or each injection as a separate experiment with different initial conditions.

3. Blank subtraction – blank experiments can be performed (identically to actual experiments except without enzyme or substrate; see **Blank subtraction**) –for determination of start-points for data analysis.
4. The kinetic trace(s) can now be fit in order to extract thermodynamic and kinetic information (see **ITC kinetics fitting scripts**).

SUPPLEMENTARY REFERENCES

1. Copeland RA. *Kinetics of Single-Substrate Enzyme Reactions*. John Wiley & Sons, Inc. (2002).

2. Schwartz PA*, et al.* Covalent EGFR inhibitor analysis reveals importance of reversible interactions to potency and mechanisms of drug resistance. *Proc Natl Acad Sci USA* **111**, 173-178 (2014).

3. Copeland RA, Pompliano DL, Meek TD. Drug-target residence time and its implications for lead optimization. *Nat Rev Drug Discov* **5**, 730-739 (2006).

4. Copeland RA. *Kinetics of Single-Substrate Enzyme Reactions*. John Wiley & Sons, Inc. (2002).

5. Callan OH, So O-Y, Swinney DC. The Kinetic Factors That Determine the Affinity and Selectivity for Slow Binding Inhibition of Human Prostaglandin H Synthase 1 and 2 by Indomethacin and Flurbiprofen. *J Biol Chem* **271**, 3548-3554 (1996).

6. VenäLäInen Jarkko I*, et al.* Slow-binding inhibitors of prolyl oligopeptidase with different functional groups at the P1 site. *Biochem J* **382**, 1003-1008 (2004).

7. Kuzmič P, Solowiej J, Murray BW. An algebraic model for the kinetics of covalent enzyme inhibition at low substrate concentrations. *Anal Biochem* **484**, 82-90 (2015).

8. Strelow JM. A Perspective on the Kinetics of Covalent and Irreversible Inhibition. *SLAS DISCOVERY: Advancing Life Sciences R&D* **22**, 3-20 (2016).

9. Jia Y, Kumar A, Patel SS. Equilibrium and Stopped-flow Kinetic Studies of Interaction between T7 RNA Polymerase and Its Promoters Measured by Protein and 2-Aminopurine Fluorescence Changes. *J Biol Chem* **271**, 30451-30458 (1996).

10. Horwitz EM, Jenkins WT, Hoosein NM, Gurd RS. Kinetic identification of a two-state glucagon receptor system in isolated hepatocytes. Interconversion of homogeneous receptors. *J Biol Chem* **260**, 9307-9315 (1985).

11. Dill KA, Bromberg S. *Molecular driving forces : statistical thermodynamics in biology, chemistry, physics, and nanoscience*. Garland Science (2011).

12. Di Trani JM, Moitessier N, Mittermaier AK. Measuring Rapid Time-Scale Reaction Kinetics Using Isothermal Titration Calorimetry. *Anal Chem* **89**, 7022-7030 (2017).

13. Todd MJ, Gomez J. Enzyme Kinetics Determined Using Calorimetry: A General Assay for Enzyme Activity? *Anal Biochem* **296**, 179-187 (2001).

14. Bakker AV, Jung S, Spencer RW, Vinick FJ, Faraci WS. Slow tight-binding inhibition of prolyl endopeptidase by benzyloxycarbonyl-prolyl-prolinal. *Biochem J* **271**, 559-562 (1990).

15. Box GEP, Hunter WG, Hunter JS. *Statistics for experimenters : an introduction to design, data analysis, and model building*. Wiley (1978).
